# The impacts of viral infection and subsequent antimicrobials on the microbiome-resistome of growing pigs

**DOI:** 10.1186/s40168-022-01312-0

**Published:** 2022-08-04

**Authors:** Tara N. Gaire, Carissa Odland, Bingzhou Zhang, Tui Ray, Enrique Doster, Joel Nerem, Scott Dee, Peter Davies, Noelle Noyes

**Affiliations:** 1 Department of Veterinary Population Medicine (VPM), College of Veterinary Medicine, University of Minnesota, Saint Paul, Minnesota USA; 2 Pipestone Veterinary Services, Pipestone, Minnesota USA; 3 State Key Laboratory of Agricultural Microbiology, College of Animal Sciences and Veterinary Medicine, Huazhong Agricultural University, Wuhan, 430070 China; 4 Pipestone Applied Research, Pipestone, Minnesota USA

**Keywords:** Antimicrobial resistance, Microbiome, Metagenomics, Swine, Porcine reproductive, Respiratory syndrome

## Abstract

**Background:**

Antimicrobials are used in food-producing animals for purposes of preventing, controlling, and/or treating infections. In swine, a major driver of antimicrobial use is porcine reproductive and respiratory syndrome (PRRS), which is caused by a virus that predisposes infected animals to secondary bacterial infections. Numerous antimicrobial protocols are used to treat PRRS, but we have little insight into how these treatment schemes impact antimicrobial resistance (AMR) dynamics within the fecal microbiome of commercial swine. The aim of this study was to determine whether different PRRS-relevant antimicrobial treatment protocols were associated with differences in the fecal microbiome and resistome of growing pigs. To accomplish this, we used a metagenomics approach to characterize and compare the longitudinal wean-to-market resistome and microbiome of pigs challenged with PRRS virus and then exposed to different antimicrobial treatments, and a group of control pigs not challenged with PRRS virus and having minimal antimicrobial exposure. Genomic DNA was extracted from pen-level composite fecal samples from each treatment group and subjected to metagenomic sequencing and microbiome-resistome bioinformatic and statistical analysis. Microbiome-resistome profiles were compared over time and between treatment groups.

**Results:**

Fecal microbiome and resistome compositions both changed significantly over time, with a dramatic and stereotypic shift between weaning and 9 days post-weaning (dpw). Antimicrobial resistance gene (ARG) richness and diversity were significantly higher at earlier time points, while microbiome richness and diversity were significantly lower. The post-weaning shift was characterized by transition from a *Bacteroides*-dominated enterotype to *Lactobacillus-* and *Streptococcus-*dominated enterotypes. Both the microbiome and resistome stabilized by 44 dpw, at which point the trajectory of microbiome-resistome maturation began to diverge slightly between the treatment groups, potentially due to physical clustering of the pigs. Challenge with PRRS virus seemed to correspond to the re-appearance of many very rare and low-abundance ARGs within the feces of challenged pigs. Despite very different antimicrobial exposures after challenge with PRRS virus, resistome composition remained largely similar between the treatment groups. Differences in ARG abundance between the groups were mostly driven by temporal changes in abundance that occurred prior to antimicrobial exposures, with the exception of *erm*G, which increased in the feces of treated pigs, and was significantly more abundant in the feces of these pigs compared to the pigs that did not receive post-PRRS antimicrobials.

**Conclusions:**

The fecal microbiome-resistome of growing pigs exhibited a stereotypic trajectory driven largely by weaning and physiologic aging of the pigs. Events such as viral illness, antimicrobial exposures, and physical grouping of the pigs exerted significant yet relatively minor influence over this trajectory. Therefore, the AMR profile of market-age pigs is the culmination of the life history of the individual pigs and the populations to which they belong. Disease status alone may be a significant driver of AMR in market-age pigs, and understanding the interaction between disease processes and antimicrobial exposures on the swine microbiome-resistome is crucial to developing effective, robust, and reproducible interventions to control AMR.

Video Abstract

**Supplementary Information:**

The online version contains supplementary material available at 10.1186/s40168-022-01312-0.

## Background

Porcine reproductive and respiratory syndrome (PRRS) is the most economically significant disease of the US swine production, estimated to cost the industry $664 million annually [1]. Losses accrue primarily through production deficits related to decreased reproductive and growth performance, as well as morbidity and mortality impacts [2]. Pigs infected with PRRS virus (PRRSv) show clinical signs of respiratory disease, while sows used for breeding may also exhibit reproductive failure. Additionally, PRRSv infections can increase susceptibility to bacterial infections [3–6], and co-infection with multiple bacterial pathogens is common in afflicted pigs [7, 8]. As a consequence of such respiratory co-infections, antimicrobial treatments are crucial for reducing the severity of clinical disease and minimizing morbidity and mortality [9]. Indeed, respiratory disease is the primary reason that the US swine producers utilize injectable, in-water and in-feed antimicrobials [10], and in particular critically important antimicrobials [11].

While critical for animal health, antimicrobial use can have unintended consequences, including alterations to the microbiome. In-feed antimicrobials and metals have been associated with differences in the fecal microbial community of commercial swine [12], although other reports showed no difference in the microbiomes of swine raised under organic versus conventional settings [13]. Early-life injection with tulathromycin has been shown to alter piglets’ gut microbiome composition and diversity in the short-term [14], but results from longitudinal studies have reported relatively minor long-term effects of antimicrobials on the swine microbiome [15–17]. These heterogeneous findings from the published literature could be due to the highly dynamic nature of the microbiome in growing swine [18–20], as well as the many confounding factors that can significantly shape host-associated microbiomes [21].

Antimicrobial use can also have the negative consequence of selecting for AMR bacteria within the gut of the exposed pigs. Again, the evidence for this is mixed. Multi-year observations of phenotypic AMR in *Escherichia coli* isolates obtained from diseased pigs in Germany demonstrated some correlation between reductions in use and prevalence of some (but not all) AMR phenotypes [22]. Farm-level swine studies have shown associations between antimicrobial use and increases in phenotypically resistant *E. coli*, but only for specific antimicrobials and specific resistance patterns [23]. Differences in antimicrobial dosage, duration, and route did not significantly alter AMR levels in fecal *E. coli* in nursery pigs with diarrhea [24]*.* Systematic reviews of this topic report a high level of between-study heterogeneity and overall low study quality [25, 26]. Furthermore, antimicrobial treatment protocols for PRRS-afflicted pigs vary widely within the USA, and there is very little literature regarding how different protocols may impact AMR in the swine population.

The vast majority of reports about use-resistance associations in swine utilize indicator bacteria such as *E. coli* or *Enterococcus* as a proxy for AMR dynamics within the bacterial population. However, AMR has been shown to be driven in large part by the underlying microbiome [27, 28], and AMR can emerge in pathogens via transfer from fecal commensals [29–31]. Another way to measure AMR is through the use of culture-independent methods such as PCR and/or metagenomic sequencing of the total DNA, both of which have been used to study use-resistance associations in swine. Again, however, findings are ambiguous. For example, antimicrobial use has been shown to significantly increase overall AMR gene (i.e., resistome) abundance in growing pigs [32]; however, other studies reported no such effect [33, 34]. These conflicting observations may be driven by the confounding impact of the underlying microbiome, which has been strongly correlated with resistome abundance and composition in growing pigs [12].

While PRRS is a viral disease, it has been shown to impact the fecal bacterial population of infected pigs, and these impacts seem to be strain- and severity-dependent [35]. PRRSv infection has been associated with a less diverse fecal microbiome [7], and pigs with the worst clinical outcomes due to PRRSv exhibited lower post-infection fecal microbiome diversity (i.e., a lower number of unique microbial families) compared to pigs with the best clinical outcomes [36]. Conversely, pigs with higher microbiome diversity at the time of PRRSv infection had better clinical outcomes compared to pigs with lower microbiome diversity [37]. The impact of PRRSv infection on the fecal microbiome has been reported to be stronger than the impact of other factors such as dietary levels of soy isoflavones [38]. However, these results were based on sequencing and analysis of the 16S rRNA gene and thus were restricted to describing the taxonomic classification of bacteria and archaea.

Given increasing evidence that PRRS drives both antimicrobial use and host-associated microbiome dynamics, it is important to better understand how PRRS and subsequent antimicrobial exposures may impact bacterial populations and AMR in afflicted pigs. Despite the importance of these four interrelated factors (i.e., PRRSv infection, antimicrobial exposures, AMR, and microbial shifts), there is scant literature to support a theoretical framework for how they may interact within commercial pig populations. Therefore, the objective of this longitudinal viral challenge study was to characterize and compare the wean-to-finish fecal microbiome-resistome of pigs with and without PRRS and subsequently exposed to two different antimicrobial treatment protocols. We hypothesized that (1) challenge with PRRSv would induce a change in the fecal microbiome of infected pigs and (2) different post-PRRS antimicrobial treatments would be associated with differences in the fecal resistome.

## Methods

### Study population and facilities

This study was approved by the Institutional Animal Care and Use Committee (IACUC) of Pipestone Veterinary Services, number 2018-2. Study piglets were sourced from a single high-health commercial sow facility with confirmed negative status for PRRSv, influenza A virus of swine, and porcine epidemic diarrhea virus. Piglets did not receive any antimicrobials while in the sow facility, and the piglets’ sows had not received any antimicrobial treatment after placement in the farrowing rooms prior to parturition and lactation. Selected piglets had remained with their dam from farrowing through weaning, and no piglets were fostered onto those dams.

At weaning (23 days of age), three piglets from each of 36 litters were haphazardly selected for enrollment in the study, with care taken to avoid low-viability piglets. The three enrolled piglets within each litter were randomly assigned to one of three treatment groups using a random number generator, with the treatment group designated by colored ear tags. A total of 108 weaned pigs (*N*=36 per treatment group) were included in the study. On weaning day, all enrolled piglets were commingled and transported into a newly built Biosafety-level 2 research facility located in southwestern Minnesota, which had not previously housed any animals. On arrival at the facility, all piglets were weighed, visually assessed for clinical signs of illness by a veterinarian and animal health personnel, and then moved into one of three different rooms, based on treatment group assignment. Each room contained 12 pens, separated by solid walls to prevent cross-contamination of solid material between the pens. Three pigs were placed into each pen, for a total of 12 pens and 36 pigs per treatment group. Each room in the facility was equipped with negative pressure ventilation with mechanical filters (minimum efficiency reporting value of 16, Camfil-Farr, Stockholm, Sweden) to filter all incoming and outgoing air. All study personnel and pig caretakers followed strict biosecurity measures, including showering and changing of all clothing when moving between rooms. When entering and exiting pens within a room, all study personnel and caretakers changed boots and gloves.

All pigs in all treatment groups were fed the same diets, which included zinc at 3000 ppm for the first 11 days post weaning (dpw), then at 2250 ppm from 12 to 20 dpw, and finally at 100 ppm for the remainder of the study to meet nutritional needs. Copper was included at 225 ppm for the first 21 dpw, then at 162 ppm for the remainder of the study. All pigs were individually identified and treatments were recorded throughout the trial. Individual Pig Care (IPC) scoring was conducted by trained personnel as a measure of morbidity and animal welfare status [39]. IPC scores were calculated using an A-B-C scoring system previously described by Zoetis where the scores refer to severity and duration of disease [40]. For all pigs in all treatment groups, IPC scoring was done every 3 days for the first 21 days after weaning, 3 times weekly for 4 weeks after the PRRSV challenge, then once weekly until the end of the trial. All mortality was documented, and dead pigs were weighed. All surviving pigs were weighed on the day of marketing.

### Treatment groups

After a 10-day acclimation period in the research facility, all study pigs (*N*=108) received modified live PRRSv vaccine (Ingelvac PRRS® MLV, Boehringer Ingelheim Vetmedica Inc., 2 mL/pig) 10 days post-weaning (dpw) to reduce the severity of clinical illness in the pigs subsequently challenged with PRRSv. If individual pigs presented with non-PRRS-related clinical disease warranting antimicrobial treatment at any point during the trial, they were administered either penicillin G procaine (PPG) or lincomycin HCl via intramuscular injection.

The 36 pigs in room A (“Minimal” group) were considered the control group. This group was not challenged with PRRSv and did not receive any antimicrobial treatments other than by injection of individual pigs as described above. The 72 pigs in rooms B and C were challenged with 2×10^3.5 TCID50 of PRRSv 1-7-4 field isolate via the intramuscular route at 44 dpw, as previously described [9]. On days 49–53 dpw, pigs in rooms B and C were administered tilmicosin phosphate (Pulmotil® AC, 250 mg/mL, Elanco) in the drinking water via a water medication device, with a target concentration of 200 parts per million (ppm). At 51 dpw, all 36 pigs in room C (“Intensive” group) were administered 5.0 mg/kg of ceftiofur crystalline free acid (EXCEDE® for Swine, Zoetis) via intramuscular injection. In room B (“Moderate” group), pigs were administered 5.0 mg/kg of ceftiofur crystalline free acid via intramuscular injection if indicated based on clinical signs. On days 57–70 dpw, all pigs in the “Intensive” group were administered chlortetracycline (400 g/ton to deliver 22mg/kg body weight) in combination with tiamulin (35 g/ton to deliver 2mg/kg body weight) in the feed for 14 days per label requirements. The details of experimental design and treatment protocols are presented in Table 1, with further details available at [41]. Average daily gain of pigs in each treatment group was also recorded and analyzed to test for differences among treatment groups using one-way analysis of variance (ANOVA).

**Table 1:** Experimental design of control and treatment protocols for PRRSv challenge and antimicrobial treatments

Treatment group^a^	PRRSv challenge	Antimicrobial exposures			
		Individual pigs	In-water	In-feed	UDD^b^
Minimal (*N*=36)	None/negative	As-needed to treat disease (penicillin G procaine or lincomycin HCl)	None	None	15
Moderate (*N*=36)	Experimentally-infected (2×10^3.5 TCID50 of a PRRSv 1-7-4 field isolate, intramuscularly)	As-needed to treat disease (penicillin G procaine or ceftiofur crystalline free acid)	Pulmotil® AC (tilmicosin phosphate, 250 mg/mL) for 5 days, starting 5 days post-PRRSv challenge	None	238
Intensive (*N*=36)	Experimentally infected (2×10^3.5 TCID50 of a PRRSv 1-7-4 field isolate, intramuscularly)	EXCEDE® (ceftiofur crystalline free acid, 5 mg/mL) to every pig, starting 7 days post-PRRSv challenge	Pulmotil® AC (tilmicosin phosphate, 250 mg/mL) for 5 days, starting 5 days post-PRRSv challenge	Chlortetracycline (400g/ton) and tiamulin, (35g/ton) for 14 days, starting 13 days post-PRRSv challenge	946

^a^All groups received modified live PRRSv vaccine (Ingelvac PRRS® MLV, Boehringer Ingelheim Vetmedica Inc., 2mL/pig) 10 days post-weaning

^b^Used daily doses were calculated as the total number of pig-days of treatment summed across all active ingredients; chlortetracycline and tiamulin were defined as a combined product as one of the treatments [41]

### Sample collection 

Fecal samples were collected from individual pigs per rectum using a gloved finger. Gloves were changed between pigs. Fecal samples were collected from each pig at 6 time points: (1) just prior to weaning and transport to the BSL-2 facility (0 dpw, “weaning/transport”), (2) the day before PPRSv vaccination (9 dpw, “pre-PRRSv vaccine”), (3) the day prior to PPRSv challenge (44 dpw, “pre-PRRSv challenge”), (4) 5 days after PPRSv challenge and prior to antimicrobial exposures (49 dpw, “pre-AMU start”), (5) after all antimicrobial exposure had ended (79 dpw, “AMU end”), and (6) just prior to marketing (149 dpw, “market”). Individual fecal samples were placed into a sterile Whirl-Pak bag and stored immediately on ice for transport to the laboratory, where they were stored at −80°C until further processing.

### Pooling and processing of fecal samples

Individual fecal samples were thawed on ice and then combined by pen within each time point (*N*=216 composite samples total). To achieve equivalent input amounts of raw feces, total composite sample weight was targeted at 0.30 g using an electronic balance. Therefore, if 2 pigs were in a pen on a given sample collection day (i.e., if a pig had died), 0.15 g of each sample was mixed; if all 3 pigs were sampled on a given collection day, then 0.10 g of feces from each pig was mixed. The PowerSoil Pro kit (Qiagen, Germany, Catalog number 47014) was used to extract DNA from each composited sample, using the QiaCube Connect (Qiagen, Germany) according to the manufacturer’s protocol. Briefly, fecal samples were placed into PowerBead Pro tubes containing 800 uL of CD1 solution. Then, tubes containing the fecal material were vortexed using the Mini Beadbeater^TM^ (2200 rpm, 20 s per cycle) three times, with a 30-s pause in-between each beating interval to prevent overheating. After centrifugation at 16,000 g for 2 min, 600 μL of supernatant was transferred into the second position of the rotor adapter and further steps were performed in the automated method on the Qiacube Connect, with 12 composite samples extracted per run. DNA concentration was determined fluorometrically on the Qubit® 4.0 (Thermo Fisher Scientific, Germany). From each composite sample of extracted DNA, 100 ng was used for library preparation with Qiaseq FX DNA Library Kit (Qiagen, Germany, Catalog number 180475), following manufacturer instructions. Quality and quantity of libraries were assessed using TapeStation (Agilent Technologies 4200) and the Qubit® 4.0, respectively. Libraries were pooled and sequenced on 5 lanes of the Illumina NovaSeq 6000 with S4 cell chemistry, targeting a depth of 50M 2×150bp paired-end reads per library.

### Sequencing and data processing

To perform resistome analysis, sequencing reads were aligned to the MEGARes v2.0.0 ARG database using the default settings of AmrPlusPlus v2.0 [42]. Briefly, low-quality and adapter-contaminated reads were removed using Trimmomatic [43], and host-associated sequences were removed by aligning the trimmed sequences to the reference *Sus scrofa* genome using Burrows-Wheeler-Alignment (BWA) version 0.6.2 [44]. Non-host reads were then aligned to MEGARes using BWA, and the resulting SAM file was parsed with both Samtools [45] and the ResistomeAnalyzer. Low-coverage ARGs were removed by imposing a minimum gene fraction of 80%, i.e., 80% of the nucleotides within each gene had to be covered by at least one read in order to be included in downstream analyses. Microbiome taxonomic composition was determined by classifying high-quality, non-host sequences using the Kraken 2 standard database [46], with default settings as implemented in the AMRPlusPlus pipeline. Prior to descriptive and statistical analyses, both ARG and microbial counts were normalized using cumulative sum scaling (CSS) with a default percentile of 0.5 [47] to account for potential differences in sequencing depth. Normalized counts at the gene and species levels were aggregated to higher levels of the MEGARes and Kraken 2 hierarchical ontologies, respectively. The MEGARes hierarchy includes the group, mechanism, class and type levels, while Kraken 2 uses Linnean hierarchy, i.e., phylum, class, order, family, genus, and species levels.

### Statistical analysis of read depth and host DNA abundance

To assess differences in sequencing depth, we used linear mixed multivariable models to assess the number of raw and non-host reads as a function of sequencing pool (1-5), treatment group (minimal, moderate, and intensive), and time point (weaning/transport, pre-PRRSv vaccine, pre-PRRSv challenge, pre-AMU start, AMU end, and market).

### Resistome and microbiome diversity analysis

Analyses were performed to compare the composite fecal microbiome and resistome dynamics between treatment groups and time points. Sequence features (i.e., ARGs or microbial taxa) present in <1% of samples were discarded prior to estimating alpha and beta diversity and prior to statistical testing.

Alpha diversity metrics including richness (observed number of features), Shannon’s diversity, and Pieulou’s evenness [48] were calculated at the phylum, class, and genus levels for the microbiome, and at the class, mechanism and group levels for the resistome. Subsequently, mixed-effects linear models as implemented in *lme4* within the *lmer* package were constructed to test for differences in alpha-diversity indices by treatment group and time point and their interaction, with pen ID as a random effect. The same model specifications were also used to test for associations between the study variables and resistome count as an outcome (i.e., number of reads aligned to ARGs).

Bray-Curtis (BC) dissimilarity distances were calculated from CSS normalized counts [49] and then used to perform non-metric multidimensional scaling (NMDS) for both the microbiome and resistome, using the “metaMDS” function in the *vegan* R package (Jari Oksanen, 2019). Ordination fit was assessed using stress values, and in cases in which stress was ≥0.2, NMDS was repeated by increasing the *trymax* parameter, i.e., the maximum number of random starts used to search for a stable solution, until a stress value of <0.2 was obtained. Differences in NMDS ordination between treatment groups and time points were tested by permutational multivariate analysis of variance (PERMANOVA) as implemented in the “adonis” function in *vegan*, using 999 permutations [50]. The significance of sample grouping was also evaluated using analysis of similarities (ANOSIM) [51]. The statistical significance was considered at an alpha of 0.05, and in the case of statistical significance at the omnibus level, post hoc pairwise comparisons were conducted using the *pairwise.adonis* function. To test for homogeneity of multivariate dispersion between treatment groups and time points (i.e., as measured using deviation from the centroids), the *betadisper* function was utilized. Post hoc ANOVA was performed when the dispersions were significantly different between the groups. Ordination results were visualized using ggplot2 [52]. A circular dendrogram of resistome composition based on BC distances was generated and annotated using the Interactive Tree of Life tool [53].

### Procrustes analysis

In order to measure the correlation between ARG-level resistome composition and genus-level microbiome composition between treatment groups, we performed Procrustes analysis using the *vegan* package in R. Principal coordinates analysis (PCoA) was performed on Euclidean distances, and the symmetric Procrustes correlation coefficients, and corresponding *P* values, were obtained using the *protest* and *procrustes* functions in *vegan*.

### Log-fold change in feature abundance

In order to identify features with differential abundance between treatment groups and time points, we utilized multivariate zero-inflated Gaussian mixture models as implemented in the *fitZig* function *metagenomeSeq*. PenID was entered into all models as a random effect using the *useMixedModel* flag. To reduce spurious findings due to very low-prevalence features, we restricted differential abundant testing to only features that had at least 50% prevalence within each time point-treatment group combination, i.e., a feature had to be present in at least 6 of 12 composite fecal samples from each treatment group at each time point in order to be included in differential abundance testing. Pairwise differences in abundance of features between treatment groups were measured as log-fold changes using the *makeContrasts* function in the *limma* package, with Benjamini-Hochberg adjustment for multiple comparisons. Differential abundance testing was performed at multiple levels of the MEGARes and Kraken 2 hierarchies for resistome and microbiome analysis, respectively.

### Analysis of enterotypes and resistotypes based on Dirichlet multinomial mixtures

To identify entero- and resisto-types based on microbiome and resistome distributions at the group and genus levels, respectively, we utilized Dirichlet-multinomial mixtures (DMMs) as implemented in the *DirichletMultinomial* package [54]. The Laplace appropriation (i.e., lowest Laplace score) [55] was used to calculate model fit and determine the optimal number of components (i.e., clusters). The proportion of samples occupying a DMM cluster by time point and treatment group was determined, and the progression of samples through each DMM per time point was determined using DMM transition models [56] and visualized using *plot.igraph* (*igraph* package, R). A chi-square test was performed to determine relationships between enterotypes and resistotypes.

## Results

### Morbidity, mortality, and antimicrobial treatments

All study pigs were assessed for clinical signs of illness throughout the study, using a standardized Individual Pig Care (IPC) scoring system. Of the 108 study pigs, 98.1% (106/108) completed the study, with two pigs in the intensive group euthanized due to severe clinical signs at 8 and 13 days post-PRRSv challenge (IPC score “C”). Results of the clinical observations were reported in detail in a companion study and were consistent with the occurrence of mild PRRSv disease in the challenged pigs [41]. Briefly, mild (IPC score A) and moderate (IPC score B) signs of clinical disease were more common in the challenged groups, which also had a slower average daily gain (0.93 versus 0.89 g per day, respectively, *P* = 0.05) than did the unchallenged control pigs.

In the minimal treatment group (i.e., the group not challenged with PRRSv), over the course of the study a total of 5 pigs required individual antimicrobial treatments based on clinical illness. Specifically, 4 pigs received intramuscular (IM) penicillin G procaine at a dose of 33,000 IU/kg, once per day for 3 days, and 1 pig received IM lincomycin HCl at a dose of 11 mg/kg once daily for 3 days. No in-feed or in-water antimicrobials were administered to any pigs in the minimal group. In addition to the predetermined post-PRRSv antimicrobial protocols described in the “Methods” section, some pigs in the moderate and intensive groups needed non-PRRSv-related individual antimicrobial treatments due to clinical indications. In the moderate and intensive groups, respectively, 3 pigs and 1 pig received IM penicillin G procaine at a dose of 33,000 IU/kg, once per day for 3 days. In the moderate group, 7 pigs necessitated individualized treatment with IM ceftiofur crystalline free acid after the PRRSv challenge, based on clinical signs. Details of treatments are presented in Table 1 and described elsewhere [41].

### Sequencing depth and quality 

Shotgun metagenomic sequencing generated a total of 13.9B paired-end reads with an average of 64.5M per sample (range: 67.1K–127.6M) (Additional file 1: Figure S1, Additional file 2: Dataset S1) across all composite fecal samples (*N*=216). To evaluate potential differences in sequencing depth, we used linear mixed models that included sequencing pool, time point, and treatment group and their interaction, with pen ID as a random effect. Based on model results, the interaction of time point and the treatment group was significantly associated with differences in raw read count (*P* = 0.01). Specifically, raw read counts for the intensive group samples collected at the end of AMU were significantly higher than raw read counts for the moderate group samples collected just prior to PRRSv challenge (*P*=0.02) as well as prior to AMU (*P*=0.03). There was no statistically significant difference in the number of reads between sequencing pools.

Across all samples, <1% of reads (mean: 0.25%, range: 0.13–3.5%) were filtered due to low quality, and the mean per-sample Phred score for raw reads was 35.7 (range: 34.1–36.0). The proportion of host (i.e., *Sus scrofa*) DNA in each sample was variable with a mean of 36% (range: 10–67% with the exception of one sample that yielded ~97% host DNA) (Additional file 1: Figure S2). As with raw read count, the non-host read counts also varied significantly by the interaction of time point and treatment group (*P*= 0.008). Specifically, non-host read counts were significantly higher in the minimal group samples collected at pre-PRRSv vaccination than the read counts obtained from both the moderate and intensive group samples collected at weaning/transport (*P* = 0.0004 and *P*=0.007, respectively) and pre-PRRSv challenge (*P*=0.003 and *P*=0.04, respectively) and from the moderate group samples collected at pre-AMU and AMU-end (*P*=0.0003 and *P*=0.032, respectively). In addition, the non-host read counts in the samples collected from the minimal group at pre-PRRSv vaccination were significantly higher than the counts obtained from the minimal group samples collected at pre-AMU start (*P*=0.004) and at market (*P*=0.012). Based on these results, we used CSS normalization to account for differences in read depth.

### Overall microbiome-resistome composition

**Fig. 1:**
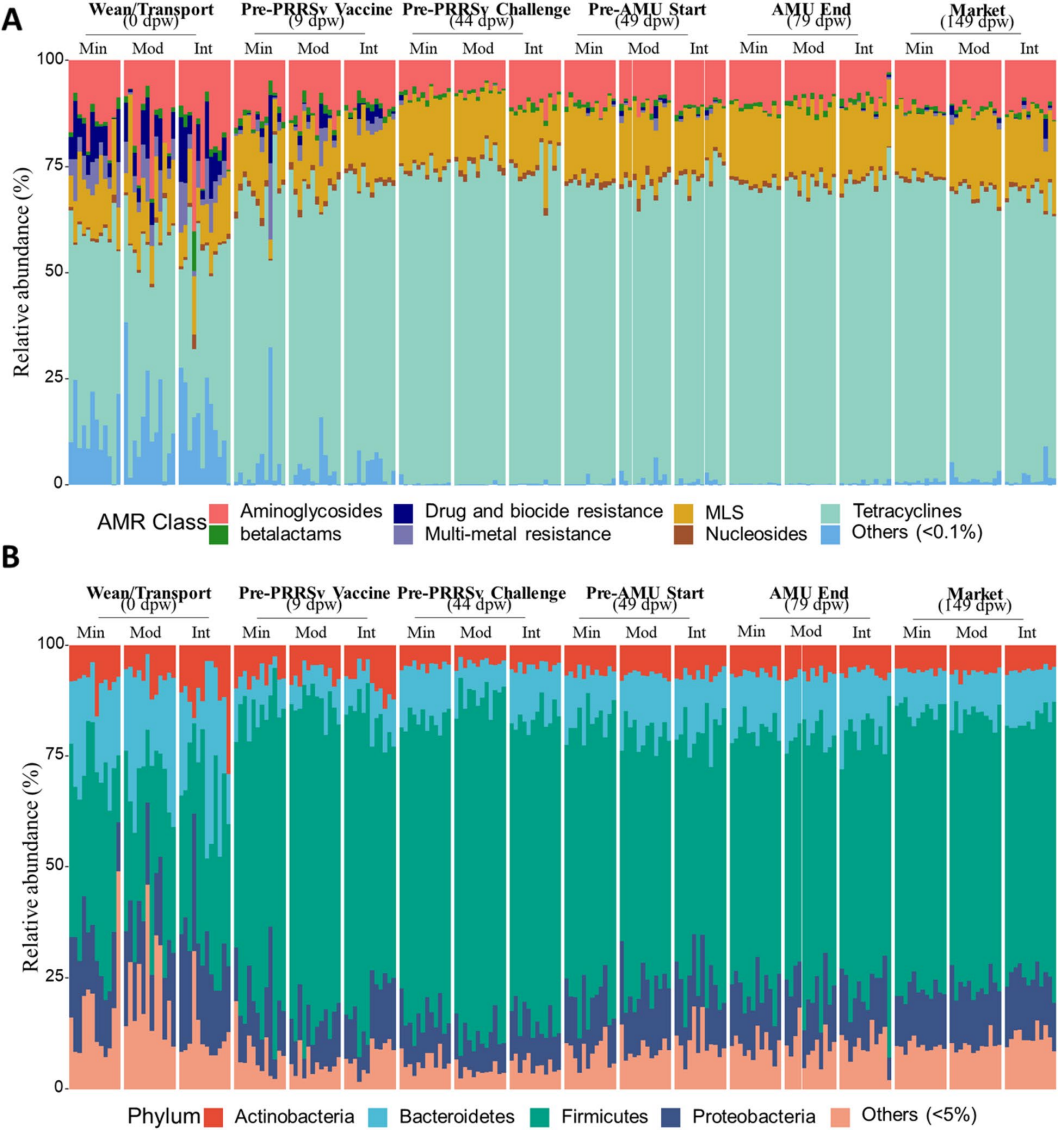
100% stacked graphs depicting a relative abundance of antimicrobial resistance (AMR) classes (**A**) and microbial genera (**B**), grouped by time point and treatment group (Min = Minimal; Mod = Moderate; Int = Intensive). AMR classes with <0.1% relative abundance and taxa phyla with <5% relative abundance across all samples were grouped as “Others”. Relative abundance was based on alignment counts normalized using cumulative sum scaling (CSS)

Non-host reads were aligned to the MEGAres 2.0 and Kraken 2.0 databases for resistome and microbiome analysis, respectively. Across all samples, ~0.23% of non-host reads aligned to ARGs within the MEGARes database. We identified 331 unique ARGs across all 216 fecal samples, which represented 88 AMR mechanisms known to confer resistance to 35 different classes of antimicrobials. Across all samples, the most abundant resistance type was drug resistance (93.8% of all ARG sequences), and only 3.0% and 2.4% of sequences originated from ARGs that confer resistance to metals and multiple compounds, respectively (Additional file 1: Figure S3). Across all treatment groups, the most abundant AMR classes were tetracycline (64.80%), followed by macrolide, lincosamide, and streptogramin B (MLS) (14.80%), and aminoglycoside (11.37%) (Fig. 1A). Tetracycline resistance ribosomal protection proteins were the most abundant AMR mechanism type (59.6% of all ARG-aligned reads), followed by 23S rRNA methyltransferases (5.88%), aminoglycoside O-phosphotransferases (5.54%), aminoglycoside O-nucleotidyltransferases (5.11%), tetracycline resistance MFS efflux pumps (4.58%), lincosamide nucleotidyltransferases (4.27%), and MLS resistance MFS efflux pumps (3.83%). Class A *β*-lactamase ARGs were relatively rare (0.88%). Within the tetracycline class, the most abundant ARG groups were *tet*(W), *tet*(Q), *tet*(O), and *tet*(40); within the MLS class, *mef*(A), *lnu*(C), *erm*(F), and *erm*(B); and within the aminoglycoside class, *ant*(6), *aph*(2)-d-prime, *aph*(3)-d-prime, and *ant*(9) groups.

Of all non-host reads across all samples, an average of 12.75% were classified taxonomically using Kraken 2 (range 10.44–17.71%). We identified 10,967 taxa across all 216 composite fecal samples. The vast majority of classified reads originated from bacteria (96.40%). After filtering sparsely represented taxa, 8528 taxa remained, comprising 41 phyla, 79 classes,179 orders, 472 families, 1642 genera, and 6784 microbial species. Of the total non-host reads across all fecal samples, 13.0%, 12.01%, 11.92%, 11.31%, 11.10%, and 10.30% were classified at the phylum, class, order, family, genus, and species levels, respectively. For the reads that mapped to the Kraken 2 database, 3.47%, 5.82%, 6.53%, 11.29%, 12.24%, and 19.80% were unclassified at the phylum, class, order, family, genus, and species level, respectively. Overall, bacteria from the phylum *Firmicutes* comprised the largest proportion of classified reads (58.49%), followed by *Proteobacteria* (12.82%) and *Bacteroidetes* (12.39%), and *Actinobacteria* (6.59%) (Fig. 1B). *Bacilli*, *Clostridia*, *Bacteroidia*, *Gammaproteobacteria*, and *Actinobacteria* were the most abundant classes. Similarly, at the genus level, *Lactobacillus*, *Streptococcus*, *Clostridium*, *Bacteroides*, *Faecalibacterium*, and *Prevotella* were the most abundant across all fecal samples. *Lactobacillus amylovorus*, *Lactobacillus reuteri*, *Faecalibacterium prausnitzii*, *Megasphaera elsdenii*, and *Lactobacillus johnsonii* were the most abundant species detected across all samples.

### The fecal microbiome-resistome shifted rapidly and consistently after weaning and then largely stabilized by 44 dpw

**Fig. 2:**
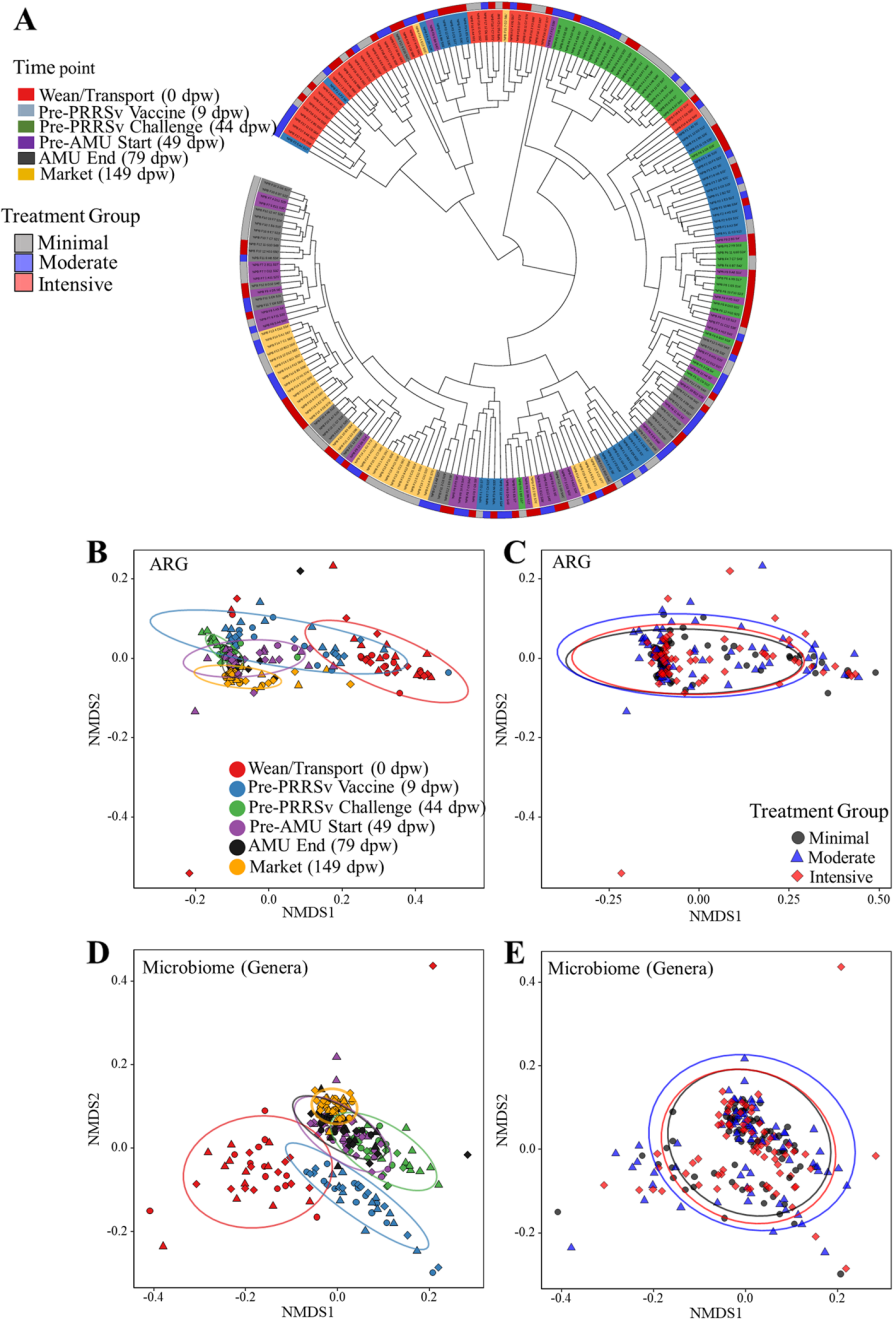
Sampling time point is associated with significant differences in fecal resistome and microbiome composition. **A** Dendrogram showing linkage clustering of Bray-Curtis (BC) dis(similarities) of fecal resistome composition at the ARG level, colored by time point and treatment group. Non-metric multidimensional scaling (NMDS) ordination based on BC (dis)similarities (stress=0.076) by **B** time point (ANOSIM*—*analysis of similarities *P*=0.001, PERMANOVA*—*permutational multivariate analysis of variance *R*^2^=53.9%, *P*=0.001) and **C** treatment group (ANOSIM *P*=0.07, PERMANOVA *R*^2^<1%, *P*=0.22). NMDS ordination based on BC (dis)similarities (stress=0.10) of microbial genera by **D** time point (ANOSIM *P*=0.001, PERMANOVA *R*^2^=42.6%, *P*=0.001) and **E** treatment group (ANOSIM, *P*=0.32, PERMANOVA *R*^2^<1%, *P*=0.11). Ellipses indicate the 95% confidence interval for distance from the centroids of each respective group of samples

**Fig. 3:**
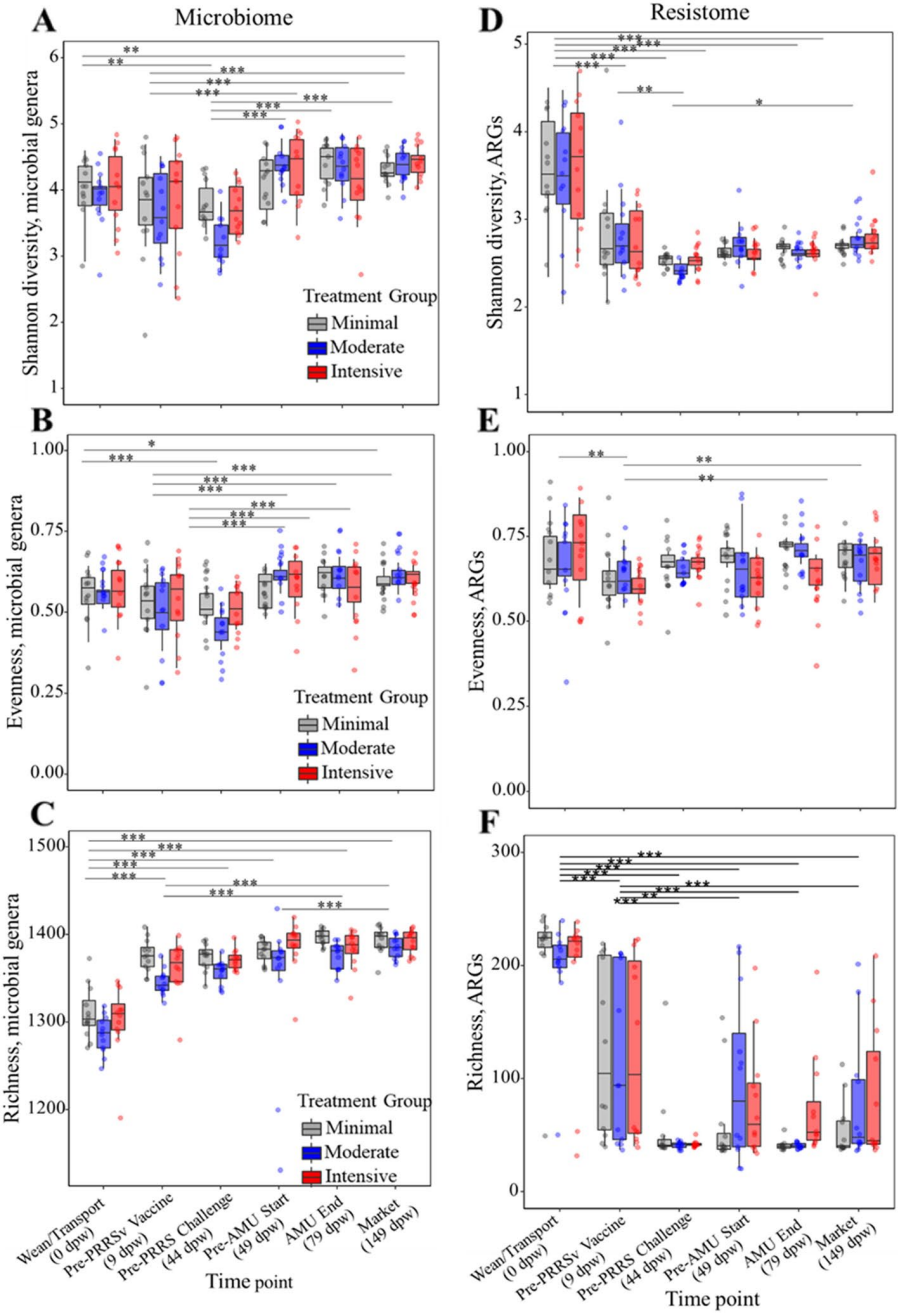
Resistome and microbiome alpha-diversity. Genus-level Shannon’s diversity (**A**), Pielou’s evenness (**B**), and richness (**C**). ARG group-level Shannon’s diversity (**D**), Pielou’s evenness (**E**), and richness (**F**). Horizontal black lines indicate pairwise comparisons between timepoints with statistically significant differences at the *P* < 0.01 (*), *P* < 0.001 (**), and *P* < 0.0001 (***), based on generalized linear mixed modeling

Both the microbiome and resistome shifted significantly between weaning and 9 dpw, as measured using nearly all ecological metrics. Non-metric multidimensional scaling (NMDS) analysis showed that the overall microbial community composition of the wean/transport samples clustered separately from all subsequent time points (Fig. 2D, Additional file 1: Figure S4), and PERMANOVA statistical testing revealed a significant difference in the microbiome of samples collected at weaning versus 9 days later (pairwise PERMANOVA *P* < 0.001). The alpha-diversity of the fecal microbiome also shifted rapidly, characterized by significantly increased richness by day 9 (pairwise *P* < 0.0001), and significantly increased Shannon’s diversity and Pielou’s evenness by day 44 (both pairwise *P* < 0.001, Fig. 3A–C). Thus, the rapid post-weaning shift was characterized by the addition of unique taxa in the first 9 days, followed by a more even distribution of those taxa by day 44.

Over 80% of the fecal microbiome in pre-weaning samples was comprised of *Proteobacteria*, *Firmicutes*, *Bacteroidetes*, and *Actinobacteria* phyla. After weaning and transport to the research facility, the relative abundance of these taxa showed marked deviation with sharp decreases in the relative abundance of *Bacteroidetes* (~21 to 8%) and *Proteobacteria* (~19 to 11%), and a nearly 2-fold increase in the relative abundance of *Firmicutes* (35 to 67%) (Fig. 1B). Results of differential abundance testing on a phylum-by-phylum basis across all samples confirmed these observations. In total, 15 phyla (mean abundance ≥ 3, log fold change—LogFC ≥ ± 1) were found to be significantly differentially abundant between the samples collected at weaning and the samples collected 9 days later, i.e., prior to PRRSv vaccination. Of these 15 differentially abundant phyla, 11 had higher abundance in the samples taken at 9 dpw while 3 phyla (i.e., *Bacteroidetes*, *Synergistetes*, and *Lentisphaerae*) had lower abundance. After these first two time points, the microbiome largely stabilized and there were relatively few significant differences in abundance of phyla over time. These results remained largely consistent when we investigated time-dependent dynamics for each treatment group separately. In the minimal, moderate, and intensive groups, 7, 11, and 10 phyla were significantly differentially abundant between weaning and 9 dpw, respectively; and this was the largest time-dependent shift observed for each treatment group (Additional file 1: Figure S5A, Additional file 2: Dataset S2). The relative abundance of the *Synergistetes* phylum decreased in all treatment groups during the first 9 days of the trial. After the 9 dpw time point, the abundance of most phyla remained stable in all treatment groups, with the exception of some minor shifts in the moderate group after PRRSv challenge and after antimicrobial exposures (Additional file 1: Figure S5A, Additional file 2: Dataset S2).

When comparing abundances of bacterial taxa between treatment groups at each time point, there were relatively few statistically significant differences. For example, at weaning, there was no significant difference in abundance of any phyla between the moderate and minimal groups, and only the abundance of *Lentisphaerae* and *Synergistetes* phyla were significantly lower in the intensive group than minimal and moderate groups, respectively. At pre-PRRSv vaccination, 3 phyla were significantly differentially abundant, with all three significantly lower in the moderate versus the minimal group. Following PRRSv vaccination, there was no significant difference in abundance of phyla between treatment groups at any time point with the exception of *Lentisphaerae*, which was in lower abundance in the moderate versus the minimal group at the pre-PRRSv challenge time point (Additional file 1: Figure S5B, Additional file 2: Dataset S3). This pattern of between-group taxa-level abundance was also reflected in genus-level analysis (Additional file 1: Figure S6A-B, Additional file 2: Dataset S3). Together, these results demonstrated that the most dramatic and consistent microbiome shift occurred in the 9 days after weaning and transport.

**Fig. 4:**
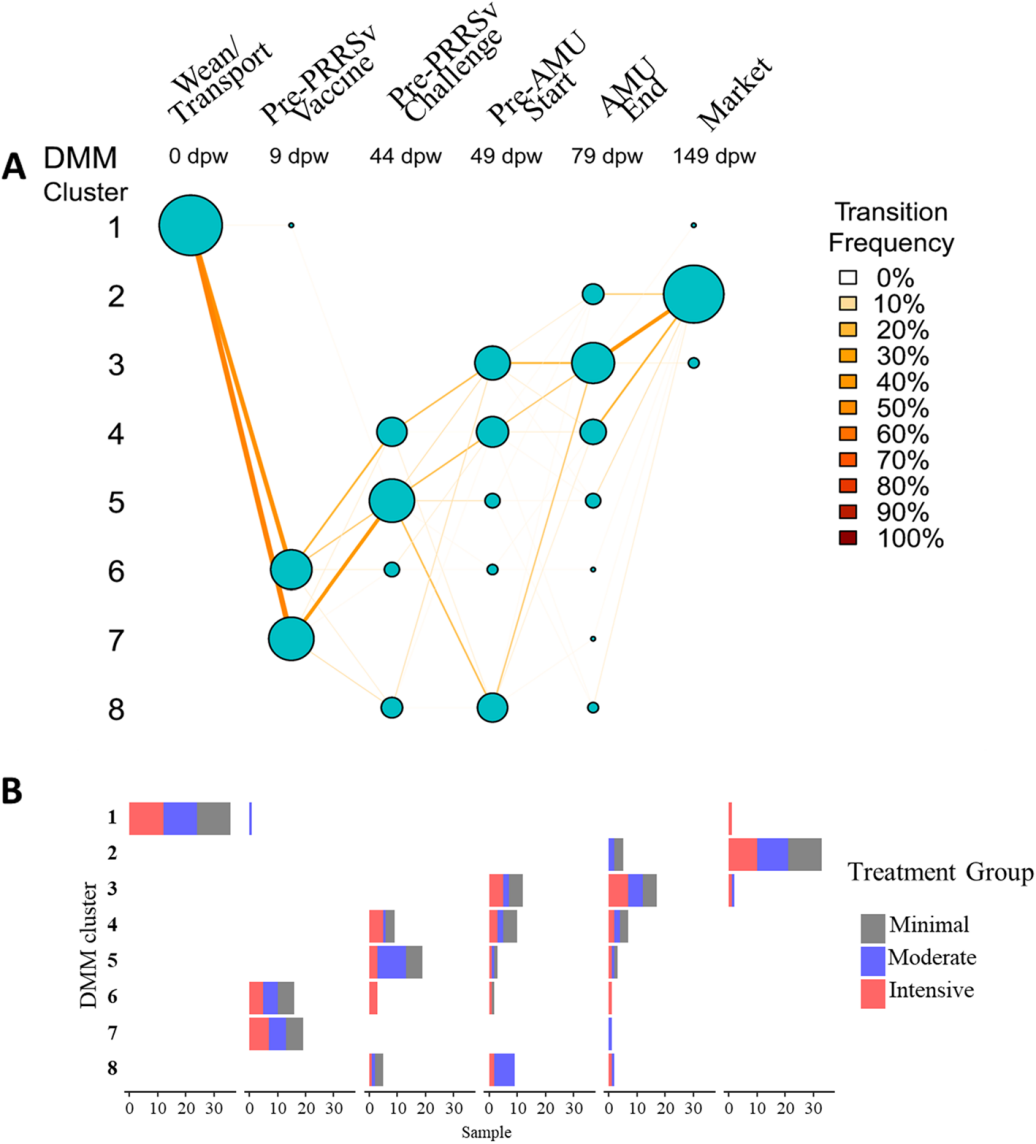
Enterotype dynamics over time and between treatment groups. Dirichlet multinomial mixture (DMM) models of genus-level microbiome alignments were used to assign samples into one of eight clusters based on the lowest Laplace approximation. **A** Transitional model showing assignment of fecal samples to DMM cluster (*y*-axis) and stratified by time point (*x*-axis). The size of circles is proportional to the number of samples contained in each DMM cluster, and edges are weighted by transition frequency. **B** Stack bar of cluster stratified by the treatment group (colored)

To further investigate microbiome transitions from weaning to market, we applied Dirichlet multinomial mixture (DMM) modeling to the microbiome count matrices at the genus level. Across all samples, eight clusters (i.e., enterotypes) were identified as the optimal number based on the lowest Laplace approximation (Additional file 1: Figure S7A). Stratifying the samples by enterotype assignment over time, we observed that all 36 composite samples collected at the pre-wean time point belonged to enterotype 1 (Fig. 4A), which was characterized by a dominance of the genus *Bacteroides* (Additional file 1: Figure S8). By the second sampling time point (9 dpw), all composite samples had transitioned to enterotypes 6 and 7, characterized by a dominance of *Lactobacillus* and *Streptococcus*, and a much smaller proportion of *Bacteroides*. This shift was observed for samples taken from all treatment groups (Fig. 4B).

A similar rapid and dramatic shift was observed in the resistome data, with clear differences in ARG composition of wean/transport samples compared to all other time points (Fig. 2A–C; class and mechanism level Additional file 1: Figure S9). However, the alpha-diversity shifts were opposite in direction from the microbiome shifts. Specifically, resistome richness, evenness, and Shannon’s diversity all decreased after the first time point (Fig. 3D–F), whereas in the microbiome data all of the indices increased during the same time period. Additionally, the resistome shift was completed more quickly than the microbiome shift, with significant differences in all metrics occurring between days 0 and 9 post-weaning (Fig. 3D–F). In addition to the progressive change in resistome diversity and composition, the number of reads aligning to ARGs dropped significantly between day 0 samples and day 44 samples (*P*=0.03 based on linear mixed modeling, Additional file 1: Figure S10). However, there was no significant association between resistome counts and treatment group or the interaction of treatment group and time point.

**Fig. 5:**
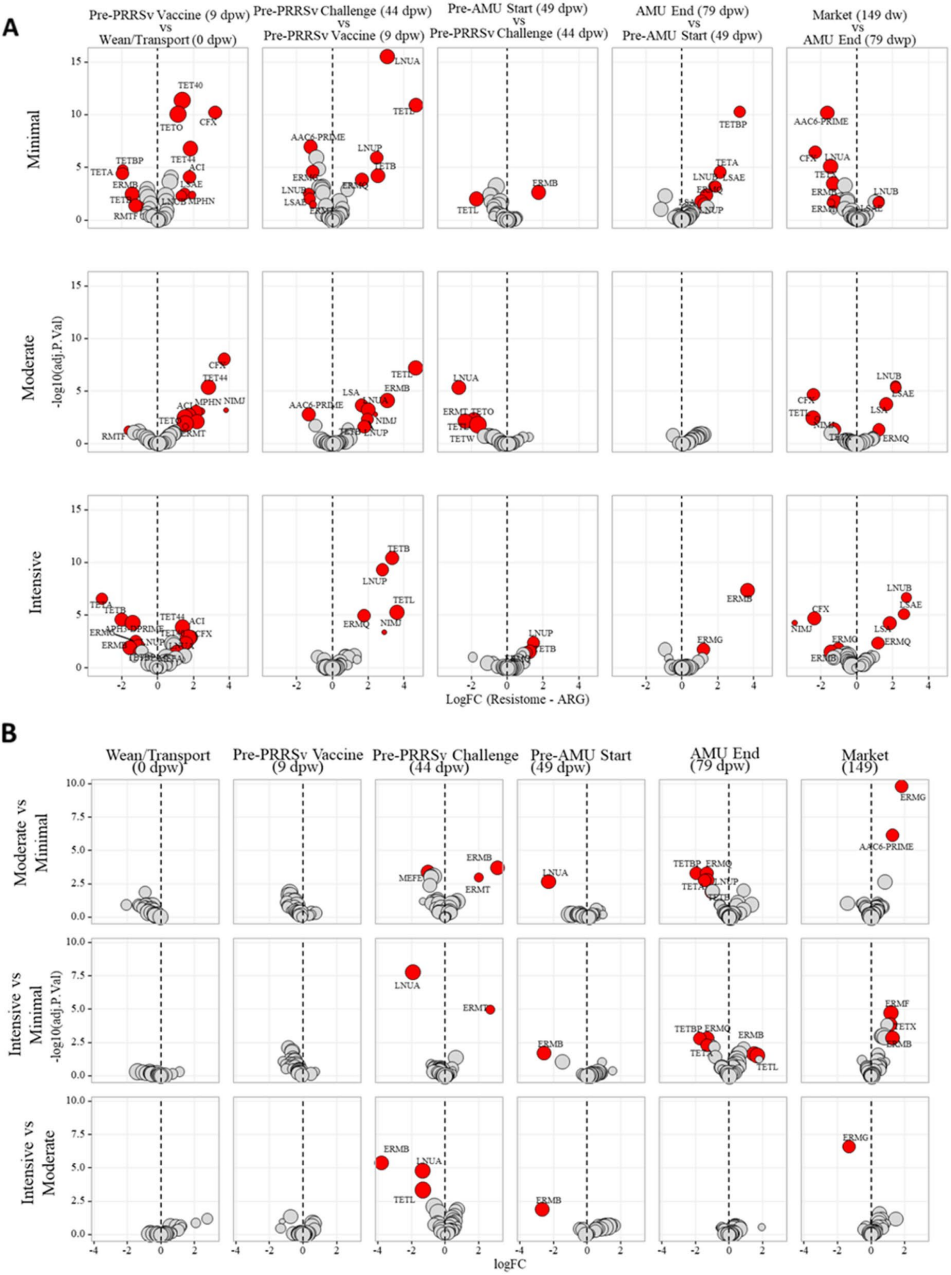
Log-fold change (logFC, *x*-axes) of ARG features **A** each treatment group (minimal, moderate, and intensive) comparing sequential sampling time points (i.e., positive logFC values indicate higher abundance in the later time point compared to the earlier time point) and **B** between treatment groups (minimal, moderate, and intensive) for each time point. Each dot represents the ARG feature in the group, and red dots represent ARG that are significantly different between comparison groups (i.e., logFC≥±1, BH adjusted *P*< 0.05)

Weaning- and transport-associated shifts in the resistome were characterized by a relative increase in the dominance of tetracycline ARGs, with concomitant decreases in the relative abundance of ARGs that confer resistance to multiple metals and to both antimicrobial drugs and biocides (Fig. 1A). Additionally, the pre-weaning resistome contained a relatively high proportion of diverse yet low-abundance ARGs, which were absent from the resistome data by day 44 post-weaning (Fig. 1). These shifts were observed in differential abundance analysis, which showed a significant increase in abundance of *tet*(W) (a tetracycline resistance ribosomal protection protein) from day 0 to day 9 samples, accompanied by significant decreases for 15 unique AMR classes, 24 unique mechanisms, and 12 unique ARGs (Fig. 5A, Additional file 2: Dataset S5). As with the microbiome, the vast majority of statistically significant changes in ARG abundance occurred from day 0 to day 9 post-weaning (Additional file 2: Dataset S5), again reflecting the rapid and dramatic shift that occurred immediately after weaning and transport of the pigs.

**Fig. 6:**
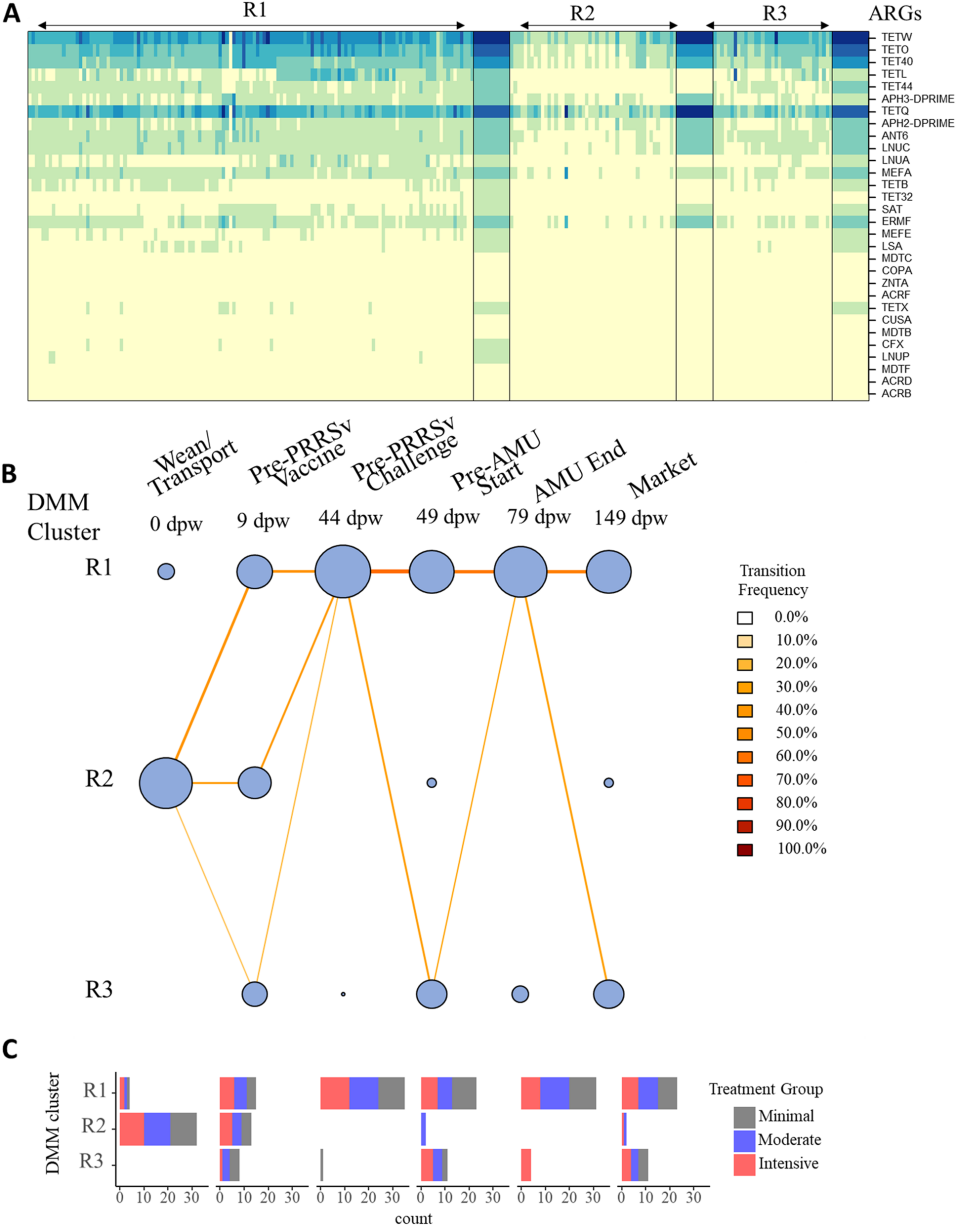
Dirichlet multinomial mixtures (DMM) samples into one of three clusters from the entire resistome composition at ARG level based on the lowest Laplace approximation. **A** Heat map showing the abundance (square root transformed) of the 30 most dominant ARG per DMM cluster, **B** transitional model showing the progressive change of sample through DMM cluster/resistotypes per each sampling point from wean-to-market across all treatments, and **C** clusters stratified by treatment group (colored). Time points are on the *x*-axis, and resistotype is represented on the *y*-axis. The size of the circle is proportional to the number of samples contained in each DMM cluster, and nodes are colored and edges are colored by transition frequency

Considering the stereotypic progression of microbiome enterotypes that we observed (Fig. 4), we performed DMM on the ARG group-level count matrices. In contrast to the microbiome, the ARG data from the 216 composite fecal samples were best clustered into 3 distinct resistotypes based on lowest Laplace approximation (Additional file 1: Figure S7B). Resistotype 1 was the most prevalent and represented nearly ~60% of the samples, while resistotypes 2 and 3 represented 23% and 16%, respectively. While resistotype 1 and 2 both contained relatively high representation of *tet*(W), *tet*(O), tet(40), and *tet*(Q), resistotype 1 also contained relatively high abundance of *tet*(L), *tet*(44), *lnu*(A), *tet*(B), *tet*(32), and *sat*, which were all virtually lacking in resistotype 2 (Fig. 6A). Nearly all of the samples obtained at weaning belonged to resistotype 2, while nearly all of the samples had transitioned to resistotype 1 by 44 dpw (i.e., just prior to PRRSv challenge in the moderate and intensive groups, Fig. 6B, C).

### Temporal shifts in the microbiome and resistome far outweighed the impacts of viral challenge and antimicrobial exposures

The impact of time point consistently outweighed the impact of treatment group across all comparisons made. PERMANOVA statistical testing demonstrated that time point explained the majority of the observed variation in the microbiome and resistome (*R*^2^ = 42% and 54%, both *P* = 0.001), while treatment group explained less than 1% of the variation in both the microbiome and resistome, and this was not statistically significant (*P* = 0.11 and 0.20, respectively). Treatment group was not significantly associated with differences in richness, Shannon’s diversity or Pielou’s evenness in either the microbiome or the resistome data.

Feature-by-feature differential abundance testing demonstrated that the vast majority of changes in abundance occurred over time (Additional file 1: Figure S6A, Additional file 2: Dataset S4 as opposed to between treatment groups Figure S6B). In total, the abundance of 317 genera changed significantly over time, compared to only 67 genera that exhibited significantly different abundance between the treatment groups at any time point (Additional file 2: Dataset S3, reporting genera with overall mean abundance >3 and logFC of ≥ ± 1). A similar pattern was observed in the resistome, with 21 ARGs changing significantly in abundance over time, compared to only 11 exhibiting significant differences between the treatment groups at any time point (Additional file 2: Dataset S5-S7). These findings demonstrate the dominance of temporally-driven microbiome-resistome dynamics, which far outweighed the impacts of viral infection and antimicrobial exposures.

### Treatment group differences in the microbiome and resistome emerged prior to PRRSv challenge

The pigs in each treatment group were managed nearly identically until PRRSv challenge. Despite this consistency in management, the fecal resistomes and microbiomes exhibited signals of divergence in the samples taken just prior to PRRSv challenge at 44 dpw. Up until this time point, there were no significant differences in differential abundance of any ARG groups between any of the treatment groups (Fig. 5B). However, in the 44 dpw samples, there were several differences between the treatment groups, despite the fact that they had been managed nearly identically prior to this time point. Specifically, *erm*(T) was significantly more abundant in the moderate and intensive groups compared to the minimal group (logFC= 1.9, adjusted *P*=0.001; logFC=2.7, *P*<0.001, respectively). *erm*(B) was significantly higher in the moderate compared to both the intensive and minimal groups (logFC=3.1, *P*=0.0001, LogFC=3.8, *P* <0.001, respectively). *lnu*(A) was significantly lower in the intensive compared to both the moderate and minimal groups (logFC= -1.9, *P*<0.001, logFC= -1.4, *P*<0.001 respectively). *tet*(L) was significantly lower in the Intensive compared to the moderate group (logFC= −1.3, *P*=0.0004). And finally, *mef*(E) was significantly lower in the moderate compared to the minimal group (logFC= −1.1, *P*=0.0003). These differences were relatively small in magnitude compared to the total resistome composition and thus did not result in differences in resistotype between the treatment groups, i.e., all of the pens of pigs at 44 dpw belonged to resistotype 1 (Fig. 6B).

A similar pattern of divergence between treatment groups was seen in the microbiome comparisons. At 0 and 9 dpw, there were barely any significant differences in genus abundance between any of the treatment groups (Additional file 1: Figure S6B). The lack of treatment group differences at these time points was particularly striking given the massive shift occurring in the populations during these time periods (Additional file 1: Figure S6A). This suggests that the impacts of weaning, transport, maturation, and commingling that occurred were incredibly consistent between the treatment groups. However, by 44 dpw, numerous genera were differentially abundant between the groups (Additional file 1: Figure S6B). These feature-by-feature differences in abundance at 44 dpw contributed to differences in enterotype assignment at the same time point, with pens distributed across enterotypes 4, 5, 6, and 8 (Fig. 4A). Most of the pigs in the moderate group belonged to enterotype 5, which was characterized by a relative predominance of *Lactobacillus* and *Streptococcus*. Pens in the minimal treatment group were distributed evenly between enterotypes 4, 5, and 8, while intensive pens belonged to enterotypes 4, 5, and 6. This differential distribution of enterotypes between the treatment groups at 44 dpw contrasted to the very consistent and even distribution in the previous time points (i.e., 0 and 9 dpw, Fig. 4B), again indicating that the fecal microbiome shifted dramatically yet remarkably consistently across all treatment groups between 0 and 9 dpw, but then started to diverge based on treatment group membership.

### PRRSv infection was associated with an increase in rare yet diverse ARGs

**Fig. 7:**
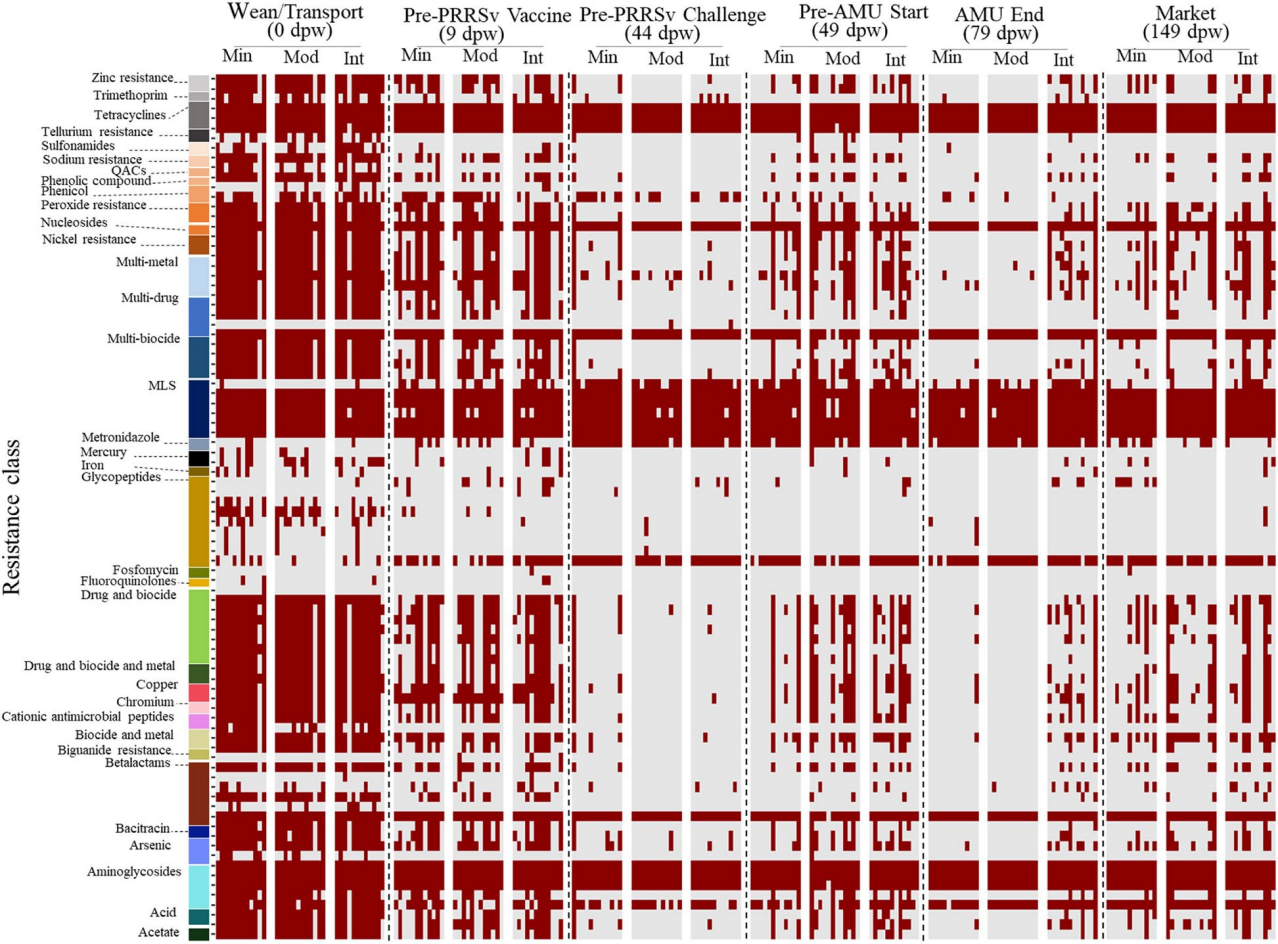
Binary heatmap (red=present, gray=absent) of resistance mechanisms for each resistance class by treatment group for each sampling time point (list of each mechanism by AMR class is presented in Additional file 1: Table S1)

Although time point dominated the patterns of microbiome and resistome change that were observed in this population, PRRSv infection was associated with some transient differences between the treatment groups. Specifically, fecal resistome richness 5 days after PRRSv challenge was higher for some of the pens in the moderate and intensive groups, compared to all of the pens in the minimal group (Fig. 3F). This increase in richness was due primarily to the addition of numerous low-abundance ARGs within the metagenomic data obtained from these pens (Fig. 7), which can also be seen in the decreased resistome evenness for many of the moderate and intensive samples taken post-challenge (Fig. 3E). The increased resistome variability within the challenged groups was also observed in the distribution of beta-dispersion for the resistome ordination, which varied significantly between time points, with higher dispersion being observed after the PRRSv challenge compared to the pre-PRRSv challenge (*P*<0.05) (Additional file 1: Figure S11A).

The rare and low-abundance ARGs that appeared in the resistome of the moderate and intensive groups 5 days post-challenge included ARGs that confer resistance to metals including zinc, nickel, mercury, iron, copper, chromium, sodium, arsenic, and tellurium; resistance to biocides including phenolic compounds, peroxide, acids, and acetate; and resistance to antimicrobial drugs including cationic antimicrobial peptides, trimethoprim, glycopeptides, betalactams, and bacitracin. For a full list of these ARGs, see Additional file 1: Table S2.

In addition to the between-treatment group dynamics of rare and low-abundance ARGs, there were several differences observed for more abundant ARGs. There was a significantly lower abundance of *erm*(B) in the post-PRRSv samples collected from the intensive group compared to both the moderate and minimal groups (logFC = −2.7 and −2.6, adjusted *P* = 0.01 and 0.02, respectively, Fig. 5B). This difference may have been driven by a temporal increase in the abundance of *erm*(B) within both the moderate and minimal groups that occurred prior to PRRSv challenge (Fig. 5B). Post-PRRSv samples from the moderate group also had significantly lower abundance of *lnu*(A) compared to samples from the minimal group. This difference was driven by a significant decrease in the abundance of *lnu*(A) in the moderate group that occurred during the 5 days post-PRRSv challenge (Fig. 5B). There were no other significant differences in the abundance of any ARGs between any of the treatment groups at 5 days post-challenge.

### Post-antimicrobial ARG differences between treatment groups were preceded by temporal changes in abundance that occurred independent of antimicrobial exposures

Fecal samples collected approximately 1 week after all antimicrobials had been discontinued exhibited several resistome patterns. In the moderate group, most of the rare ARGs that had appeared in the post-challenge data were no longer present, whereas they did persist in the data obtained from the intensive group (Fig. 7). This included ARGs for zinc resistance, sodium resistance, peroxide resistance, penicillin binding proteins, nickel resistance, multi-metal RND and ABC efflux pumps, multi-biocide resistance mechanisms, mercury resistance, lipid A modification, multi-compounds resistance mechanisms, copper resistance, arsenic, and acid resistance.

When investigating more prevalent ARGs (i.e., those with presence in at least 50% of samples from every treatment group at each time point, Fig. 5A), we found no statistically significant differences in abundance when comparing fecal samples taken from the moderate and intensive pigs at the end of antimicrobial administrations (Fig. 5B). However, there were 7 differentially abundant ARGs when comparing the moderate and minimal groups and 11 when comparing the intensive and minimal groups. For the former group of 7 differentially abundant ARGs, 6 were less abundant in the fecal samples collected from the moderate group compared to the minimal group (*tet*(BP), *erm*(Q), *tet*(A), *tet*(B), *lnu*(A), and *lnu*(P)), while *erm*(G) was significantly more abundant in the moderate group (logFC = 0.8, adjusted *P* = 0.009). Among the 11 differentially abundant ARGs between the intensive and minimal groups at the end of antimicrobial exposures, 6 were lower and 5 were higher in the intensive group compared to the minimal group. As with the pattern seen for the moderate group, *tet*(BP), *erm*(Q), *tet*(A), *lsa*(E), *lnu*(B), and *lnu*(P) were all significantly lower in the intensive group compared to the minimal group. However, these differences were not driven by the antimicrobial exposures themselves. Rather, they were driven by temporal increases in the abundance of these ARGs within the minimal group, which occurred contemporaneously during the time period in which the moderate and intensive groups were receiving antimicrobials (Fig. 5A). In addition to these differences, the Intensive group had significantly higher abundance of *erm*(G), *erm*(B), *erm(F)*, *cfr*, and *tet*(L) after antimicrobial administration compared to the minimal group, with logFC values ranging from 0.5 for *cfr* to 1.7 for *tet*(L) (Fig. 5A and Additional file 2: Dataset S5). Again, some of these differences were not driven by increases in abundance within the intensive group during the period of antimicrobial exposures (Fig. 5A), but rather by preceding and/or contemporaneous temporal decreases in abundance within the minimal group. For example, *tet*(L) significantly decreased in abundance from 44 to 49 dpw in the minimal group, resulting in a relatively higher abundance of this ARG within the intensive group in the post-antimicrobial samples. However, other differences in ARG abundance that we observed after antimicrobial administration were likely driven by significant increases that occurred within the intensive group during antimicrobial exposure. For example, *erm*(B) and *erm*(G) both increased significantly within the intensive group during the period of antimicrobial administration (Fig. 5A), which likely resulted in the significantly higher abundance of these ARGs in the intensive versus the minimal group at the 79 dpw time point (Fig. 5B).

The significantly increased abundance of *erm*(G) in the moderate compared to the minimal group persisted in the samples taken at market (logFC = 1.8, adjusted *P* < 0.001), and this was also significantly higher than the abundance in the Intensive group (logFC = 1.3, *P* < 0.001). In addition, the market-age samples from the moderate group had a significantly higher abundance of *erm*(F) and *aac*(6’) compared to the minimal group (logFC = 0.8 and 1.3, adjusted *P* < 0.001 and 0.002, respectively). There were no other statistically significant differences between the moderate and minimal groups at a market age. When comparing the intensive and minimal group at the same time point, we identified 9 ARGs that were significantly differentially abundant, and all of them had higher abundance in the intensive samples compared to the minimal samples, with logFC’s ranging from 0.4 for *aph*(2”) to 1.3 for *erm*(B). Finally, the intensive samples contained significantly higher abundance of *erm*(T) and *ant*(9) compared to the moderate samples at marketing (logFC = 0.8 and 0.5, adjusted *P* = 0.03 and 0.03, respectively).

We further evaluated the relationship between microbiome and resistome composition for each treatment group by a sampling point using Procrustes analysis. We did not observe significant correlations between fecal resistome and microbiome compositions with the exception of the intensive group at the pre-PRRSv challenge time point (*M*^2^=0.56, correlation=0.66, *P*=0.007) and at the end of antimicrobial administration (*M*^2^=0.49, correlation=0.71, *P*=0.005). The resistotypes were found to be associated with enterotypes (chi-square test, *P* < 0.001). Nearly 60% of the samples belonged to resistotype 1, which was represented within all enterotypes but observed most frequently in enterotypes 2, 3, 4, and 5 (Additional file 1: Figure S12A). Resistotype 2 samples had higher ARG diversity than samples assigned to the other resistotypes (Additional file 1: Figure S12B), which was mostly associated with samples that were also assigned to enterotype 1. Resistotype 3 never occurred in the same sample as enterotype 1 and was most commonly assigned to samples that also were assigned to enterotype 2.

## Discussion

### The swine fecal microbiome-resistome matures in a stereotypic manner, and pig grouping patterns can impact the maturation trajectory

We observed a rapid, consistent, and dramatic shift in the fecal microbiome of commercial pigs, which occurred within the week after they were weaned and transported to a new facility. The dramatic impact of weaning on the swine microbiome has been previously reported, and our results confirm that microbiome richness and diversity increase significantly post-weaning [57–61]. As with previous reports, we found that *Bacteroides* were predominant in the samples taken just prior to weaning [61, 62]. Using DMM enterotyping, we observed that pre-weaning samples were also characterized by a relative dominance of *Alistipes*, *Cloacibacillus*, *Escherichia*, and *Intestinimonas*. Indeed, these four genera did not reach the top 10 most prevalent genera in any of the post-weaning samples, suggesting that their relative abundance within the pre-weaning samples is a defining characteristic of the suckling piglet fecal microbiome. Previous studies of the peri-weaning microbiome also found a predominance of *Alistipes* [62].

Based on a metagenomic approach, we observed that the fecal resistome also experienced a dramatic, consistent, and rapid shift after weaning, characterized by a loss of diversity and richness and an increased predominance of tetracycline ARGs such as *tet*(Q), *tet*(O), and *tet*(W), many of which have been previously reported as dominant within the gut resistome of mature pigs raised with and without antibiotics [63], as well as in the feces of commercial pigs across 9 European countries [34]. The consistent impact of age on phenotypic and genotypic AMR in bacteria isolated from swine feces has been documented [64], although the temporal dynamics specifically around weaning have not been widely investigated. There is also very little literature regarding microbiome-wide AMR profiles in weaning piglets. Piglets experience many changes during the weaning period, including transportation, exposure to a new environment and transition from a milk- to a solid-based diet. All of these factors can alter the gut microbiome composition [65] and immune function, which can negatively impact pigs health and growth performance [66]. More research is needed to understand how these microbiome alterations at weaning shape the resistome profile, especially because young animals and humans are known to harbor diverse ARG profiles [67]; our findings support that the early-life swine fecal resistome is similarly diverse. The piglets in this study were born to sows that did not receive antimicrobials during lactation, and the piglets themselves did not receive antimicrobials prior to weaning. Therefore, the composition of the resistome at weaning was not the direct result of contemporaneous antimicrobial exposures in the pigs or their dams. The source and establishment of early-life ARGs in neonates are not well characterized, but studies in humans have found that both the mother and the built environment contribute to the ARG colonization in neonates [68]. Further investigation is needed to identify the definitive source of the diverse ARGs present in neonatal swine feces.

The juxtaposition of increasing microbiome diversity and decreasing resistome diversity after weaning is intriguing. There is strong evidence to suggest that shifts in the fecal microbiome drive shifts in the fecal resistome of maturing hosts [69]. In human neonates, an inverse correlation has been observed between microbiome alpha diversity and overall resistome burden, i.e., lower microbiome diversity was associated with a higher ARG burden [70]. After the weaning transition, both the microbiome and the resistome profiles stabilized by 44 dpw, as indicated by very tight boxplots for richness, diversity, and evenness (Fig. 3) as well as very low betadispersion (i.e., mean distance to centroid) values (Additional file 1: Figure S11). The uniformity of the post-weaning microbiome-resistome trajectory may have been driven by dietary shifts that were also uniform across the study pigs, which all received the same post-weaning diet. By day 44, most pens had transitioned to one of two dominant enterotypes and one dominant resistotype. The enterotypes at this stage were characterized by a predominance of *Lactobacillus* and *Streptococcus*, while the resistotype was characterized by a relative predominance of *tet*(L), *tet*(44), and *tet*(32). However, while the overall microbiome-resistome composition was largely stabilized by 44 dpw, it is also noteworthy that the abundances of numerous ARGs and genera were significantly different between the treatment groups (Fig. 5B, Additional file 1: Figure S6B), despite nearly identical management of the groups prior to this time point. These treatment group differences at 44 dpw contrasted markedly to the lack of differences at 0 and 9 dpw (Fig. 5B, Additional file 1: Figure S6B). Taken together, these patterns suggest that the fecal microbiome-resistome matured in a remarkably stereotypic pattern across all pigs in the early post-weaning period, but this stereotypic progression began to diverge by 44 dpw, likely driven by the strict physical separation of the treatment groups. Previous studies have shown that the microbiomes of piglets housed in the same pen are more similar to each other than to the microbiomes of piglets from other pens [71, 72], suggesting that groups of piglets develop a unique microbiome composition, presumably due to unique microbial, host, and environmental factors within the group. As observed in this and other studies, these group-level differences are typically outweighed by factors such as piglet age, but nevertheless result in detectable and significant microbiome differences between groups [73]. This dynamic may have important implications for microbiome-based interventions in livestock populations because it suggests that the impact of such interventions may differ across groups of animals, even within the same farm. This finding also emphasizes the need to account for host grouping structures in the design and execution of microbiome studies.

### PRRSv infection disrupted resistome stability and was associated with an increase in generalized stress response genes

The relative resistome stability observed at day 44 was disrupted by the PRRSv challenge, as evidenced by very wide boxplots for richness, diversity, and evenness within the challenged groups (Fig. 3D–F). The impact of the PRRSv challenge was especially notable given that all study pigs received PRRSv vaccination at 10 dpw, resulting in relatively mild post-challenge clinical disease; in naive animals with more severe clinical illness, resistome changes may be more dramatic. The increased variability that we observed in this study population indicates that the resistomes of some pens of pigs were detectably impacted by the PRRSv challenge, while others maintained a resistome more similar to that of the non-challenged group. The high amount of between-pen variability may be due to differences in viral load, clinical disease severity, or immune response to the viral challenge within individual pigs. However, we were unable to evaluate such associations due to the pooled nature of our fecal samples. An association between clinical outcomes and microbiome diversity has been reported in pigs that were co-challenged with PRRSv and porcine circovirus type 2 [36], but no literature exists regarding the mechanistic relationship between PRRSv and AMR.

The resistomes of pens that exhibited increased richness after the PRRSv challenge were characterized by numerous low-abundance ARGs that appeared within the metagenomic data. Because these ARGs were very low-abundance, we cannot discount the potential impact of sequencing depth on these results. For example, the “pre-AMU” and “AMU end” samples obtained from the Intensive group contained significantly higher numbers of raw reads compared to the other combinations of time point and treatment group. The use of CSS normalization should account for this difference. Additionally, if sequencing depth were the sole driver of these results, we would expect to observe increased richness in the intensive group only. Instead, we observed this pattern in the moderate group as well. Finally, increased sequencing depth would also likely result in additional microbiome richness due to the detection of very low-abundance taxa. However, we did not observe similar increased richness in the microbiome data obtained from post-PRRSv samples in the intensive group. Taken together, these considerations suggest that the resistome variability seen after PRRSv challenge stemmed from biological dynamics, rather than measurement bias.

A related question is whether the appearance of these low-abundance ARGs within the post-PRRSv samples was due to acquisition of the ARGs in those pens of pigs or increased abundance of the ARGs to a level that exceeded the limit of detection of the metagenomic assay. Given that these pens of pigs had been in highly biosecure rooms for 49 days at the time of post-PRRSv sample collection, it is unlikely that these ARGs represent acquisition events; there was no other potential source from which to acquire new ARGs. A more supportable hypothesis is that we detected these ARGs because their abundance within the microbiome had increased sufficiently to be detectable. The fact that these ARGs were present in the fecal metagenome of these pigs at weaning (Fig. 1) further suggests that their “re-appearance” within the data was due to increases in abundance rather than new acquisition events. ARGs can increase in abundance through several mechanisms, including replication of the bacterial genomes harboring them, and horizontal gene transfer (HGT) from donor to recipient bacterial cells. We were unable to confidently determine which process may have been driving the post-PRRSv challenge resistome dynamics due to the fragmented nature of short-read metagenomic data and the resulting inability to accurately localize AMR genes with their proper hosts [74]. We did, however, observe that a number of pens in the moderate and intensive groups shifted to a new enterotype after PRRSv infection (i.e., enterotype 8), which could indicate that underlying microbiome dynamics were driving resistome changes.

The primary ARGs that appeared within the metagenomic data of the moderate and intensive groups post-challenge included mechanisms for zinc, arsenic, and multi-metal resistance (i.e., RND and MFS efflux pumps, respectively, Fig. 7). These genes were included in the latest version of MEGARes as resistance mechanisms [42], yet they can also be characterized as acquisition-tolerance mechanisms, i.e., they allow bacteria to regulate acquisition of metals, while also increasing tolerance for excessive metal levels in the microbe’s environment [75]. The role of host-versus-pathogen metal nutrient sequestration and acquisition during pathogenic infection has been extensively studied and is known to influence pathogenicity [76]. Less is known about how commensal gut microbes regulate metals during infection events. The other ARGs that appeared within the fecal resistome of PRRSv-infected pens included mechanisms of peroxide, acetate, acid, and multi-biocide resistance. These mechanisms of resistance have primarily been studied in relation to pathogens and their ability to cause clinical disease [77, 78], and little is known about their functional or ecological role within commensal microbiomes. However, all of these ARGs could broadly be termed bacterial “stress response” mechanisms, and host infection is known to induce pathogen stress responses via changes in the physicochemical properties of the host environment [79, 80]. Therefore, one hypothesis for our resistome observations is that the PRRS viral infection caused systemic immune and inflammatory activation in the pig, which changed the microenvironment of the gut, prompting increased abundance of these stress response ARGs within the commensal microbiome.

### Exposure to high levels of antimicrobials had variable impacts on ARGs from relevant antimicrobial classes, with no evidence of large or persistent microbiome-resistome changes

We hypothesized that feces from pigs in the moderate and intensive groups would exhibit increases in MLS ARGs compared to feces from the minimal group, due to exposure to therapeutic doses of oral tilmicosin via their drinking water. Indeed, we observed significantly higher abundance of *erm*(G) in post-AMU and market samples obtained from both the moderative and intensive groups compared to the minimal group. Additionally, *erm*(F) had higher abundance in the post-AMU intensive samples and the market-age intensive and moderate samples compared to the minimal samples at the same time points. These differences were modest in magnitude, i.e., typically less than two log-fold difference in sequence counts. In a recent extensive analysis of *erm* genes, *erm*(F), and *erm*(G) were found solely in genomes from the phylum *Bacteroidetes*, and *erm*(G) was the second-most common *erm* gene obtained from metagenomic fecal samples of swine [81]*. erm*(G) has been previously associated with phenotypic resistance to macrolides in *Clostridium* isolates obtained from swine feces and is thought to be associated with mobile genetic elements that can transfer between gram-positive and gram-negative bacteria [82, 83]. This genomic plasticity may account for the increases seen in *erm*(G*)* after macrolide administration in this study. *erm*(B) and *erm*(T) were also higher in the Intensive group samples collected at market-age compared to the minimal market-age samples. However, *erm*(Q) was lower in the intensive and moderate groups compared to the minimal group after antimicrobial exposures. *erm*(Q) is not widely reported in the literature but has been characterized as the most common macrolide resistance gene in *Clostridium perfringens* [84]. Of note, an analysis of fecal *Enterococcus* isolates obtained from the pigs in this study did not identify significant differences between treatment groups in resistance to macrolide antibiotics, suggesting that the ARG-level differences identified here did not notably impact phenotypic resistance in this important group of bacteria [41]. These companion results demonstrate the different types of information that can be gleaned from culture versus metagenomic approaches and suggest that both data sources could be used in tandem to gain a more complete understanding of AMR in livestock populations.

The pigs in the Intensive group all received therapeutic doses of ceftiofur and oral chlortetracycline, and we hypothesized that this would cause increases in relevant ARGs in the feces of exposed pigs. In line with this hypothesis, we observed a significantly higher abundance of *tet*(L) in the post-AMU samples collected from the intensive group compared to the minimal group samples at the same time point, with a nearly 2-log-fold difference. *tet*(L) is a relatively under-described tetracycline efflux pump that does not seem to share a common ancestral lineage with other *tet* genes [85]. Counterintuitively, we also detected a significantly lower abundance of *tet*(A) and *tet*(BP) when comparing the same samples, with ~1.5-log-fold lower abundance in the intensive compared to the minimal group. We observed the same pattern when comparing the moderate post-AMU samples to the minimal post-AMU samples. However, these post-AMU differences were likely caused by decreasing temporal abundance of *tet*(BP) and *tet*(A) in the minimal group during the period of antimicrobial administration (Fig. 5A), rather than temporal increases within the moderate and intensive groups. These findings highlight the complexity of microbiome-resistome dynamics in rapidly growing animals and emphasize the need to account for underlying temporally driven changes in AMR.

The *tet-*ARG patterns observed after antimicrobial administration did not persist in the market-age samples. Instead, at marketing, the intensive group had significantly higher abundance of *tet*(X) and *tet*(Q) compared to the moderate group, and there were no other significant differences in *tet*-ARG abundance between any of the groups. *tet*(X) homologs can inactive numerous tetracycline formulations including tigecycline, but the level and spectrum of resistance is closely linked to single-nucleotide polymorphisms (SNPs), which are not robustly detectable in metagenomic data [86]. The distribution of *tet*(X) across microbes is not well understood, and a recent review of mobile ARGs concluded that there is not enough evidence to confidently determine the recent origin of *tet*(X) [87]*. tet*(Q) is a broadly distributed ARG identified in gram-positive and gram-negative bacteria that can competently transfer between diverse taxa [88]. The increased abundance of these ARGs in the market-age fecal samples of the intensive pigs could be due to the gradual or delayed impact of increased HGT caused by selective pressure from chlortetracycline exposure, although further study is needed to reproduce these results. There were no significant differences in abundance of any betalactam ARGs between any of the treatment groups at any post-PRRSv time point. This could be the result of overall very low betalactam ARG abundance within this study population, which is consistent with previous resistome profiles of swine feces [34, 89].

In addition to group differences in *tet* ARG abundance after antimicrobial exposures, we found that the intensive and moderate groups had significantly lower abundance of *lnu*(B)*, lnu*(A), and *lnu*(P) compared to the minimal group and significantly higher abundance of *aac6’* in the market-age samples. These ARGs confer resistance to lincosamide and aminoglycoside antimicrobials, respectively. Interestingly, we also observed phenotypic resistance to streptomycin in fecal *Escherichia coli* isolates obtained from these same study pigs [41]. However, aminoglycosides were not used in this study population, and several pigs within each treatment group received lincomycin treatments prior to PRRSv challenge. Therefore, the differences in these ARGs do not relate directly or linearly to antimicrobial exposures in this study population. Furthermore, the abundance of *lnu*(B)*, lnu*(A), and *lnu*(P) decreased significantly over time in the minimal group, which likely resulted in the between-group differences at marketing. These results highlight the dynamic nature of the fecal resistome and the corresponding fact that associations between AMU and AMR are rarely straightforward. In order to truly understand the mechanism by which antimicrobials impact AMR within microbial populations, there is need for a more sophisticated framework that takes into account ecological and evolutionary relationships, as well as common phenomena such as co-selection and HGT. Further metagenomic studies may wish to include complementary assays that can better elucidate these complex dynamics, including Hi-C [90].

An important component of this study was the sampling of pigs at marketing, i.e., the day that they were sent for slaughter. These samples have particular relevance for food safety and public health because they represent the fecal resistome of the pigs just prior to entering the food chain. Additionally, the fecal microbiome is a primary source of contamination within slaughter plants (along with the hide), and therefore, fecal ARGs could be an important source of foodborne ARGs. We found that antimicrobial exposures did not appreciably alter the AMR fecal profile of market-ready pigs, despite the fact that some of the pigs were continuously exposed to oral tilmicosin and then chlortetracycline-tiamulin for a cumulative period of 19 days and received an injection of ceftiofur. This finding has implications for antimicrobial use in the US swine herds experiencing a PRRSv challenge and suggests that antimicrobial protocols commonly used in these scenarios may not cause significantly increased levels of fecal ARGs as measured using metagenomics. This conclusion is also supported by phenotypic antimicrobial susceptibility results from the same study population, wherein we observed minor differences in the likelihood of resistant fecal *E. coli and Enterococcus spp.* isolates obtained from pigs receiving different intensities of antimicrobial exposures [41]. Previous studies have identified significant associations between high levels of macrolide exposure and increased odds of sulfamethoxazole and chloramphenicol in *E. coli* [91] and between oxytetracycline exposure and temporary increases in tetracycline-resistant fecal coliforms [24]. These studies were based on phenotypic resistance in cultured bacteria. Studies using a genotypic assay have reported more mixed results, similar to what we observed in this study. For example, Birkegård et al. found inconsistent associations between antimicrobial exposures in swine and fecal ARG levels measured using qPCR and concluded that most of the variation in ARG abundance was due to factors other than antimicrobial exposures [92]. Looft et al. found associations between in-feed tylosin and increased abundance of aminoglycoside ARGs in the feces of exposed pigs [93]. Other studies reported broader associations between antimicrobial use and resistome profiles; however, these studies were primarily ecological in nature, comparing samples across production systems, countries and years without accounting for potential confounding by management practice, environment, or host differences [34, 94]. A strength of our study was the use of identical environments and management practices for the study pigs, thus removing these as potential confounders.

In previous studies, in-feed antimicrobials have been shown to significantly alter the fecal microbiome of swine managed under highly controlled conditions [93, 95–97]. We did not observe similar changes in our study; however, it should be noted that previous studies utilized a much lower dose of tilmicosin for a longer duration, which may account for these differences in results. Inconsistent findings between antimicrobial use studies have been attributed to heterogeneous antimicrobial protocols and overall low study quality [26]. Recent reports of antimicrobial use patterns in the US swine operations also indicated a large amount of variability in protocols between systems and years, with oral tetracyclines comprising a majority of the use by weight [11]. A unique feature of the work reported here was the inclusion of antimicrobial treatment protocols that are commonly used by the US swine producers confronting respiratory health challenges, including in-water chlortetracycline and injectable third-generation cephalosporins [10]. However, replication studies are needed to confirm our results, especially given the apparent importance of group-level dynamics and the fact that we had only one physical room per treatment group. Future studies should carefully consider group-level design, as well as how this design relates to the unit of sampling and analysis. Previous work in this area has focused largely on study outcomes related to individual pathogens or host factors [98, 99], and more work is needed to understand how study design can be optimized to account for and elucidate interactions between microbial populations, their hosts, and the groups within which hosts live. Another limitation of our study was that we were not able to track AMR profiles beyond the lifecycle of a single cohort of pigs and thus could not make observations about potential multi-generational impacts of the antimicrobial exposures. Such exposures may be important in maintaining ARGs within a continuous flow of pigs [100]. Multi-generational studies would also help reveal the source and carryover of ARGs within neonatal pigs, which is an important component of understanding ARG ecology within swine herds.

## Conclusions

The fecal microbiome-resistome of growing pigs underwent a stereotypic shift driven primarily by weaning-associated changes and pig age. Exposure to PRRSv corresponded with the (re)-appearance of rare and low abundance ARGs. Despite large differences in intensity of antimicrobial exposures after PRRSv challenge, resistome composition remained largely similar between the treatment groups, with modest differences in the abundance of several ARGs, only some of which corresponded with antimicrobial exposures at the drug class level. Overall, these results suggest that viral disease itself may be a potential driver of AMR in the fecal microbiome, in addition to subsequent AMU. In order to disentangle these two potential AMR risk factors and develop targeted, evidence-based guidance on AMU in livestock populations, it is critical to improve our understanding of the mechanistic drivers of AMR in both diseased and healthy animals of various ages. Gaining this understanding requires a more detailed investigation of microbiome-wide ecological and evolutionary dynamics at different stages of disease progression and different antimicrobial protocols.

## Supplementary Information

**Additional file 1: Figure S1**. Boxplots of the raw sequence read counts by time point and treatment group (Minimal—dark grey, Moderate—blue and Intensive—red color). Horizontal lines forming each box represent the first quartile, median and third quartile, while whiskers denote 1.5x the interquartile range. **Figure S2**. Proportion of host (*Sus scrofa*) to non-host reads per sample (x-axis) by treatment groups for each time point. **Figure S3**. Relative abundance of resistance by type (drugs, biocides, multi-compounds and metals) in composite fecal samples, separated by treatment group (panels) and sampling time point (weaning/transport to market). **Figure S4**. Microbiome composition (NMDS—non metric multidimensional scaling, Bray-Curtis dissimilarity) at phylum level (stress=0.09) by A) sampling time point (ANOSIM—Analysis of similarities *P*=0.001, PERMANOVA—Permutational multivariate analysis of variance R^2^=49%, *P*=0.001). B) by treatment (ANOSIM P=0.238, PERMANOVA R^2^ <1%, *P*=0.113). C) microbiome composition at class level (stress=0.10) by sampling time point (ANOSIM P=0.001, PERMANOVA R^2^=47%, *P*=0.001). D) by treatment (ANOSIM *P*=0.388, PERMANOVA R^2^ <1%, *P*=0.167). Ellipse indicates 95% confidence interval for distance around centroids of the group. **Figure S5**. Log-fold change (logFC, x-axes) of microbial phyla for A) each treatment group (Minimal, Moderate and Intensive) comparing sequential sampling time points (i.e., positive logFC values indicate higher abundance in the later time point compared to the earlier time point) and B) between treatment groups (Minimal, Moderate and Intensive) for each sampling time point. Each dot represents a microbial phylum in the group and red dots represent genera that are significantly different between comparison groups (i.e., logFC≥±1, mean abundance ≥ 3, BH adjusted *P*< 0.05). **Figure S6**. Log-fold change (logFC, x-axes) of microbial genera for A) each treatment group (Minimal, Moderate and Intensive) comparing sequential time points (i.e., positive logFC values indicate higher abundance in the later time point compared to the earlier sampling time point) and B) between treatment groups (Minimal, Moderate and Intensive) for each time point. Each dot represents a microbial genera in the group and red dots represent genera that are significantly different between comparison groups (i.e., logFC≥±1, mean abundance ≥ 3, BH adjusted *P*< 0.05). **Figure S7**. Model fit for number of Dirichlet mixture components (DMM), K using the Laplace approximation, A) microbiome at genus level and B) resistome at ARG level. **Figure S8**. 100% stacked bar of top 10 taxa (microbial genera) within each DMM cluster. **Figure S9**. Resistome composition (NMDS, Bray-Curtis dissimilarity) at class level (stress=0.048) by A) sampling time point (ANOSIM *P*=0.001, PERMANOVA R^2^=56%, *P*=0.001). B) by treatment (ANOSIM *P*=0.487, PERMANOVA R^2^<1%, *P*=0.502). C) Resistome composition at mechanism level (stress=0.066) by sampling time point (ANOSIM P=0.001, PERMANOVA R^2^=54.8%, *P*=0.001). D) by treatment (ANOSIM *P*=0.08, PERMANOVA R^2^ <1%, *P*=0.344). Ellipse indicates 95% confidence interval for distance around centroids of the group. **Figure S10**. Number of reads aligned to resistome (i.e ARGs) (cumulative sum scaling normalized) in the MEGARes database by time point and treatment group (represented by colored dots). Horizontal lines forming each box represent the first quartile, median and third quartile, while whiskers denote 1.5x the interquartile range. Each sample is represented by a dot with horizontal jitter. **Figure S11**. Effect of sampling time point on the multivariate dispersion of A) resistome at ARG level and B) microbiome at genus level. Both resistome and microbiome beta-diversity are measured as the distance to their group centroid (using Bray-Curtis distance). **Figure S12**. Boxplot of A) Number of samples for each resistotype depicted as a function of enterotypes (microbiome DMM cluster), B) ARG diversity of treatment group for each time point according to the resistotypes. **Table S1**. List of AMR mechanisms for each AMR class presented in heatmap Fig. 7. **Table S2**. List of low abundant ARGs that were detected post-PRRSv challenge in moderate and intensive treatment groups.

**Additional file 2: Dataset S1**. Metadata for all samples presented in this study. **Dataset S2**. Results from microbiome (phylum and genus level) differential abundance testing of each treatment group, by sampling time point. **Dataset S3**. Results from microbiome (phylum and genus level) differential abundance testing between treatment group, for each sampling time point. **Dataset S4**. Results from microbiome (phylum and genus level) differential abundance testing by sampling time point (ie. all treatment group samples together). **Dataset S5**. Results from resistome (ARG level ie. AMR gene group) differential abundance testing of each treatment group, by sampling time point. **Dataset S6**. Results from resistome (ARG level ie. AMR gene group) differential abundance between treatment group, for each sampling time point. **Dataset S7**. Results from resistome (ARG level ie. AMR gene group) differential abundance by sampling time point (ie. all treatment group samples together).

## Data Availability

Sequence data generated in this study are publicly available in the NCBI SRA under BioProject number PRJNA788462. Metadata for all samples included in this study are presented in additional file 2: dataset S1.

## Abbreviations

AMR: Antimicrobial resistance
AMU: Antimicrobial use
ANOSIM: Analysis of similarities
ARG: Antimicrobial resistance gene
CSS: Cumulative sum scaling normalization
DMM: Dirichlet multinomial mixtures models
DPW: Day post-weaning
LogFC: Log-fold change
NMDS: Nonmetric multidimensional scaling
PERMANOVA: Permutational multivariate analysis of variance
PRRSv: Porcine reproductive and respiratory syndrome virus

## References

[1] Holtkamp DJ (2013). Assessment of the economic impact of porcine reproductive and respiratory syndrome virus on United States pork producers. J Swine Health Prod..

[2] Neumann EJ, Kliebenstein JB, Johnson CD, Mabry JW, Bush EJ, Seitzinger AH (2005). Assessment of the economic impact of porcine reproductive and respiratory syndrome on swine production in the United States. J Am Vet Med Assoc..

[3] Yu J, Wu J, Zhang Y, Guo L, Cong X, Du Y (2012). Concurrent highly pathogenic porcine reproductive and respiratory syndrome virus infection accelerates Haemophilus parasuis infection in conventional pigs. Vet Microbiol..

[4] Thanawongnuwech R, Brown GB, Halbur PG, Roth JA, Royer RL, Thacker BJ (2000). Pathogenesis of porcine reproductive and respiratory syndrome virus-induced increase in susceptibility to Streptococcus suis infection. Vet Pathol..

[5] Solano GI, Segalés J, Collins JE, Molitor TW, Pijoan C (1997). Porcine reproductive and respiratory syndrome virus (PRRSv) interaction with Haemophilus parasuis. Vet Microbiol..

[6] Wills RW, Gray JT, Fedorka-Cray PJ, Yoon KJ, Ladely S, Zimmerman JJ (2000). Synergism between porcine reproductive and respiratory syndrome virus (PRRSV) and Salmonella choleraesuis in swine. Vet Microbiol..

[7] Jiang N, Liu H, Wang P, Huang J, Han H, Wang Q (2019). Illumina MiSeq sequencing investigation of microbiota in bronchoalveolar lavage fluid and cecum of the swine infected with PRRSV. Curr Microbiol..

[8] XiangjinYan, Zeng J, Li X, Zhang Z, Din AU, Zhao K (2020). High incidence and characteristic of PRRSV and resistant bacterial co-infection in pig farms. Microb Pathog..

[9] Dee S, Guzman JE, Hanson D, Garbes N, Morrison R, Amodie D, Galina Pantoja L (2018). A randomized controlled trial to evaluate performance of pigs raised in antibiotic-free or conventional production systems following challenge with porcine reproductive and respiratory syndrome virus. PLoS One..

[10] USDA–APHIS–VS–CEAH. NAHMS Swine 2012 Study. https://www.aphis.usda.gov/animal_health/nahms/swine/downloads/swine2012/Swine2012_dr_PartII_revised.pdf

[11] Davies PR, Singer RS (2020). Antimicrobial use in wean to market pigs in the United States assessed via voluntary sharing of proprietary data. Zoonoses Public Health..

[12] Zhao Y, Su J-Q, An X-L, Huang F-Y, Rensing C, Brandt KK (2018). Feed additives shift gut microbiota and enrich antibiotic resistance in swine gut. Sci Total Environ..

[13] Gerzova L, Babak V, Sedlar K, Faldynova M, Videnska P, Cejkova D (2015). Characterization of antibiotic resistance gene abundance and microbiota composition in feces of organic and conventional pigs from four EU countries. PloS One..

[14] Schokker D, Zhang J, Zhang L-L, Vastenhouw SA, Heilig HGHJ, Smidt H (2014). Early-life environmental variation affects intestinal microbiota and immune development in new-born piglets. PloS One..

[15] Holman DB, Chénier MR (2014). Temporal changes and the effect of subtherapeutic concentrations of antibiotics in the gut microbiota of swine. FEMS Microbiol Ecol..

[16] Zeineldin MM, Megahed A, Blair B, Burton B, Aldridge B, Lowe J. Negligible impact of perinatal tulathromycin metaphylaxis on the developmental dynamics of fecal microbiota and their accompanying antimicrobial resistome in piglets. Front Microbiol. 2019;10:726. 10.3389/fmicb.2019.00726. 10.3389/fmicb.2019.00726 PMC6460945 31024502

[17] Zeineldin M, Megahed A, Burton B, Blair B, Aldridge B, Lowe JF (2019). Effect of single dose of antimicrobial administration at birth on fecal microbiota development and prevalence of antimicrobial resistance genes in piglets. Front Microbiol..

[18] Slifierz MJ, Friendship RM, Weese JS (2015). Longitudinal study of the early-life fecal and nasal microbiotas of the domestic pig. BMC Microbiol..

[19] Ke S, Fang S, He M, Huang X, Yang H, Yang B (2019). Age-based dynamic changes of phylogenetic composition and interaction networks of health pig gut microbiome feeding in a uniformed condition. BMC Vet Res..

[20] Han GG, Lee J-Y, Jin G-D, Park J, Choi YH, Kang S-K (2018). Tracing of the fecal microbiota of commercial pigs at five growth stages from birth to shipment. Sci Rep..

[21] Vujkovic-Cvijin I, Sklar J, Jiang L, Natarajan L, Knight R, Belkaid Y (2020). Host variables confound gut microbiota studies of human disease. Nature..

[22] Moennighoff C, Thomas N, Nienhaus F, Hartmann M, Menrath A, Merkel J (2020). Phenotypic antimicrobial resistance in Escherichia coli strains isolated from swine husbandries in North Western Germany – temporal patterns in samples from laboratory practice from 2006 to 2017. BMC Vet Res..

[23] Burow E, Rostalski A, Harlizius J, Gangl A, Simoneit C, Grobbel M (2019). Antibiotic resistance in Escherichia coli from pigs from birth to slaughter and its association with antibiotic treatment. Prev Vet Med..

[24] Græsbøll K, Damborg P, Mellerup A, Herrero-Fresno A, Larsen I, Holm A (2017). Effect of tetracycline dose and treatment mode on selection of resistant coliform bacteria in nursery pigs. Appl Environ Microbiol..

[25] Burow E, Simoneit C, Tenhagen B-A, Käsbohrer A (2014). Oral antimicrobials increase antimicrobial resistance in porcine E. coli--a systematic review. Prev Vet Med..

[26] Sargeant JM, Bergevin MD, Churchill K, Dawkins K, Deb B, Dunn J (2019). A systematic review of the efficacy of antibiotics for the prevention of swine respiratory disease. Anim Health Res Rev..

[27] Smith DR, Temime L, Opatowski L (2021). Microbiome-pathogen interactions drive epidemiological dynamics of antibiotic resistance: a modeling study applied to nosocomial pathogen control. eLife..

[28] Relman DA, Lipsitch M (2018). Microbiome as a tool and a target in the effort to address antimicrobial resistance. Proc Natl Acad Sci..

[29] Liu Y-Y, Wang Y, Walsh TR, Yi L-X, Zhang R, Spencer J (2016). Emergence of plasmid-mediated colistin resistance mechanism MCR-1 in animals and human beings in China: a microbiological and molecular biological study. Lancet Infect Dis..

[30] Cantón R, Coque TM (2006). The CTX-M beta-lactamase pandemic. Curr Opin Microbiol..

[31] Poirel L, Rodriguez-Martinez J-M, Mammeri H, Liard A, Nordmann P (2005). Origin of plasmid-mediated quinolone resistance determinant QnrA. Antimicrob Agents Chemother..

[32] Ghanbari M, Klose V, Crispie F, Cotter PD (2019). The dynamics of the antibiotic resistome in the feces of freshly weaned pigs following therapeutic administration of oxytetracycline. Sci Rep..

[33] Pollock J, Muwonge A, Hutchings MR, Mainda G, Bronsvoort BM, Gally DL (2020). Resistance to change: AMR gene dynamics on a commercial pig farm with high antimicrobial usage. Sci Rep..

[34] Munk P, Knudsen BE, Lukjancenko O, Duarte ASR, Van Gompel L, Luiken REC (2018). Abundance and diversity of the faecal resistome in slaughter pigs and broilers in nine European countries. Nat Microbiol..

[35] Argüello H, Rodríguez-Gómez IM, Sánchez-Carvajal JM, Pallares FJ, Díaz I, Cabrera-Rubio R, et al. Porcine reproductive and respiratory syndrome virus impacts on gut microbiome in a strain virulence-dependent fashion. Microb Biotechnol. 2021. 10.1111/1751-7915.13757. 10.1111/1751-7915.13757 PMC8913879 33656781

[36] Niederwerder MC, Jaing CJ, Thissen JB, Cino-Ozuna AG, McLoughlin KS, Rowland RRR (2016). Microbiome associations in pigs with the best and worst clinical outcomes following co-infection with porcine reproductive and respiratory syndrome virus (PRRSV) and porcine circovirus type 2 (PCV2). Vet Microbiol..

[37] Ober RA, Thissen JB, Jaing CJ, Cino-Ozuna AG, Rowland RRR, Niederwerder MC (2017). Increased microbiome diversity at the time of infection is associated with improved growth rates of pigs after co-infection with porcine reproductive and respiratory syndrome virus (PRRSV) and porcine circovirus type 2 (PCV2). Vet Microbiol..

[38] Smith BN, Fleming SA, Wang M, Dilger RN (2020). Alterations of fecal microbiome characteristics by dietary soy isoflavone ingestion in growing pigs infected with porcine reproductive and respiratory syndrome virus. J Anim Sci..

[39] Pineiro C (2014). Individual Pig Care program improves productive performance and animal health in nursery-growing pigs. J Swine Health Prod..

[40] Pantoja LG (2013). Impact of a Husbandry Education Program on nursery pig mortality, productivity, and treatment cost. J Swine Health Prod..

[41] Odland CA, Edler R, Noyes NR, Dee SA, Nerem J, Davies PR. Evaluation of the impact of antimicrobial use protocols in PRRS virus infected swine on phenotypic antimicrobial resistance patterns. Appl Environ Microbiol. 10.1128/AEM.00970-21. 10.1128/AEM.00970-21 PMC8752131 34644164

[42] Doster E, Lakin SM, Dean CJ, Wolfe C, Young JG, Boucher C (2020). MEGARes 2.0: a database for classification of antimicrobial drug, biocide and metal resistance determinants in metagenomic sequence data. Nucleic Acids Res..

[43] Bolger AM, Lohse M, Usadel B (2014). Trimmomatic: a flexible trimmer for Illumina sequence data. Bioinformatics..

[44] Li H, Durbin R (2009). Fast and accurate short read alignment with Burrows–Wheeler transform. Bioinformatics..

[45] Li H, Handsaker B, Wysoker A, Fennell T, Ruan J, Homer N (2009). The sequence alignment/map format and SAMtools. Bioinformatics..

[46] Wood DE, Lu J, Langmead B. Improved metagenomic analysis with Kraken 2. Genome Biol. 2019;20(1):257. 10.1186/s13059-019-1891-0. 10.1186/s13059-019-1891-0 PMC6883579 31779668

[47] Paulson JN, Stine OC, Bravo HC, Pop M (2013). Robust methods for differential abundance analysis in marker gene surveys. Nat Methods..

[48] Pielou EC (1966). The measurement of diversity in different types of biological collections. J Theor Biol..

[49] Legendre P, Gallagher ED (2001). Ecologically meaningful transformations for ordination of species data. Oecologia..

[50] Anderson MJ (2001). A new method for non-parametric multivariate analysis of variance. Austral Ecol..

[51] Clarke KR (1993). Non-parametric multivariate analyses of changes in community structure. Aust J Ecol..

[52] Wickham H, Wickham H (2009). ggplot2: elegant graphics for data analysis.

[53] Letunic I, Bork P (2021). Interactive Tree Of Life (iTOL) v5: an online tool for phylogenetic tree display and annotation. Nucleic Acids Res..

[54] Morgan M. DirichletMultinomial: Dirichlet-Multinomial Mixture Model Machine Learning for Microbiome Data. R package version 1.38.0. 2022. https://bioconductor.org/packages/release/bioc/html/DirichletMultinomial.html

[55] Holmes I, Harris K, Quince C (2012). Dirichlet multinomial mixtures: generative models for microbial metagenomics. PLOS ONE..

[56] Stewart CJ, Ajami NJ, O’Brien JL, Hutchinson DS, Smith DP, Wong MC (2018). Temporal development of the gut microbiome in early childhood from the TEDDY study. Nature..

[57] Massacci FR, Berri M, Lemonnier G, Guettier E, Blanc F, Jardet D (2020). Late weaning is associated with increased microbial diversity and Faecalibacterium prausnitzii abundance in the fecal microbiota of piglets. Anim Microbiome..

[58] Mach N, Berri M, Estellé J, Levenez F, Lemonnier G, Denis C (2015). Early-life establishment of the swine gut microbiome and impact on host phenotypes. Environ Microbiol Rep..

[59] Pieters M, Pijoan C, Fano E, Dee S (2009). An assessment of the duration of Mycoplasma hyopneumoniae infection in an experimentally infected population of pigs. Vet Microbiol..

[60] Luise D, Le Sciellour M, Buchet A, Resmond R, Clement C, Rossignol M-N (2021). The fecal microbiota of piglets during weaning transition and its association with piglet growth across various farm environments. PLoS ONE..

[61] Frese SA, Parker K, Calvert CC, Mills DA (2015). Diet shapes the gut microbiome of pigs during nursing and weaning. Microbiome..

[62] Suriyaphol P, Chiu JKH, Yimpring N, Tunsagool P, Mhuantong W, Chuanchuen R (2021). Dynamics of the fecal microbiome and antimicrobial resistome in commercial piglets during the weaning period. Sci Rep..

[63] Tunsagool P, Mhuantong W, Tangphatsornruang S, Am-In N, Chuanchuen R, Luangtongkum T, Suriyaphol G. Metagenomics of antimicrobial and heavy metal resistance in the cecal microbiome of fattening pigs raised without antibiotics. Appl Environ Microbiol. 2021;87(8):e02684–20. 10.1128/AEM.02684-20. 10.1128/AEM.02684-20 PMC8091117 33547058

[64] Gaire TN, Scott HM, Sellers L, Nagaraja TG, Volkova VV (2021). Age dependence of antimicrobial resistance among fecal bacteria in animals: a scoping review. Front Vet Sci..

[65] Guevarra RB, Lee JH, Lee SH, Seok M-J, Kim DW, Kang BN (2019). Piglet gut microbial shifts early in life: causes and effects. J Anim Sci Biotechnol..

[66] Campbell JM, Crenshaw JD, Polo J (2013). The biological stress of early weaned piglets. J Anim Sci Biotechnol..

[67] Moore AM, Patel S, Forsberg KJ, Wang B, Bentley G, Razia Y (2013). Pediatric fecal microbiota harbor diverse and novel antibiotic resistance genes. PloS One..

[68] Klassert TE, Zubiria-Barrera C, Kankel S, Stock M, Neubert R, Lorenzo-Diaz F (2020). Early bacterial colonization and antibiotic resistance gene acquisition in newborns. Front Cell Infect Microbiol..

[69] Lebeaux RM, Coker MO, Dade EF, Palys TJ, Morrison HG, Ross BD (2021). The infant gut resistome is associated with E. coli and early-life exposures. BMC Microbiol..

[70] Gibson MK, Wang B, Ahmadi S, Burnham C-AD, Tarr PI, Warner BB (2016). Developmental dynamics of the preterm infant gut microbiota and antibiotic resistome. Nat Microbiol..

[71] Choudhury R, Middelkoop A, Bolhuis JE, Kleerebezem M (2019). Legitimate and reliable determination of the age-related intestinal microbiome in young piglets; rectal swabs and fecal samples provide comparable insights. Front Microbiol..

[72] Surendran Nair M, Eucker T, Martinson B, Neubauer A, Victoria J, Nicholson B, Pieters M. Influence of pig gut microbiota on Mycoplasma hyopneumoniae susceptibility. Vet Res. 2019;50(1):86. 10.1186/s13567-019-0701-8. 10.1186/s13567-019-0701-8 PMC6819593 31661027

[73] Bian G, Ma S, Zhu Z, Su Y, Zoetendal EG, Mackie R (2016). Age, introduction of solid feed and weaning are more important determinants of gut bacterial succession in piglets than breed and nursing mother as revealed by a reciprocal cross-fostering model. Environ Microbiol..

[74] Slizovskiy IB, Mukherjee K, Dean CJ, Boucher C, Noyes NR. Mobilization of Antibiotic Resistance: Are Current Approaches for Colocalizing Resistomes and Mobilomes Useful? Front Microbiol. 2020;11:1376. 10.3389/fmicb.2020.01376. 10.3389/fmicb.2020.01376 PMC7338343 32695079

[75] Choudhury R, Srivastava S (2001). Zinc resistance mechanisms in bacteria. Curr Sci..

[76] Porcheron G, Garenaux A, Proulx J, Sabri M, Dozois C (2013). Iron, copper, zinc, and manganese transport and regulation in pathogenic Enterobacteria: correlations between strains, site of infection and the relative importance of the different metal transport systems for virulence. Front Cell Infect Microbiol..

[77] Pericone CD, Park S, Imlay JA, Weiser JN (2003). Factors contributing to hydrogen peroxide resistance in streptococcus pneumoniae include pyruvate oxidase (SpxB) and avoidance of the toxic effects of the fenton reaction. J Bacteriol..

[78] Kanjee U, Houry WA (2013). Mechanisms of acid resistance in Escherichia coli. Annu Rev Microbiol..

[79] Fang FC, Frawley ER, Tapscott T, Vázquez-Torres A (2016). Bacterial stress responses during host infection. Cell Host Microbe..

[80] Avican K, Aldahdooh J, Togninalli M, Mahmud AKMF, Tang J, Borgwardt KM (2021). RNA atlas of human bacterial pathogens uncovers stress dynamics linked to infection. Nat Commun..

[81] Choi J, Rieke EL, Moorman TB, Soupir ML, Allen HK, Smith SD, Howe A. Practical implications of erythromycin resistance gene diversity on surveillance and monitoring of resistance. FEMS Microbiol Ecol. 2018;94(4):fiy006. 10.1093/femsec/fiy006. 10.1093/femsec/fiy006 PMC5939627 29346541

[82] Wang Y, Wang G-R, Shoemaker NB, Whitehead TR, Salyers AA (2005). Distribution of the ermG gene among bacterial isolates from porcine intestinal contents. Appl Environ Microbiol..

[83] Shoemaker NB, Vlamakis H, Hayes K, Salyers AA (2001). Evidence for extensive resistance gene transfer among Bacteroides spp. and among Bacteroides and other genera in the human colon. Appl Environ Microbiol..

[84] Berryman DI, Lyristis M, Rood JI (1994). Cloning and sequence analysis of ermQ, the predominant macrolide-lincosamide-streptogramin B resistance gene in Clostridium perfringens. Antimicrob Agents Chemother..

[85] Juricova H, Matiasovicova J, Kubasova T, Cejkova D, Rychlik I (2021). The distribution of antibiotic resistance genes in chicken gut microbiota commensals. Sci Rep..

[86] Gasparrini AJ, Markley JL, Kumar H, Wang B, Fang L, Irum S (2020). Tetracycline-inactivating enzymes from environmental, human commensal, and pathogenic bacteria cause broad-spectrum tetracycline resistance. Commun Biol..

[87] Ebmeyer S, Kristiansson E, Larsson DGJ (2021). A framework for identifying the recent origins of mobile antibiotic resistance genes. Commun Biol..

[88] Leng Z, Riley DE, Berger RE, Krieger JN, Roberts MC (1997). Distribution and mobility of the tetracycline resistance determinant tetQ. J Antimicrob Chemother..

[89] Noyes NR, Weinroth ME, Parker JK, Dean CJ, Lakin SM, Raymond RA (2017). Enrichment allows identification of diverse, rare elements in metagenomic resistome-virulome sequencing. Microbiome..

[90] Stalder T, Press MO, Sullivan S, Liachko I, Top EM (2019). Linking the resistome and plasmidome to the microbiome. ISME J..

[91] Rosengren LB, Waldner CL, Reid-Smith RJ, Dowling PM, Harding JCS (2007). Associations between feed and water antimicrobial use in farrow-to-finish swine herds and antimicrobial resistance of fecal Escherichia coli from grow-finish pigs. Microb Drug Resist Larchmt N..

[92] Birkegård AC, Halasa T, Græsbøll K, Clasen J, Folkesson A, Toft N (2017). Association between selected antimicrobial resistance genes and antimicrobial exposure in Danish pig farms. Sci Rep..

[93] Looft T, Johnson TA, Allen HK, Bayles DO, Alt DP, Stedtfeld RD (2012). In-feed antibiotic effects on the swine intestinal microbiome. Proc Natl Acad Sci U S A..

[94] Mencía-Ares O, Cabrera-Rubio R, Cobo-Díaz JF, Álvarez-Ordóñez A, Gómez-García M, Puente H (2020). Antimicrobial use and production system shape the fecal, environmental, and slurry resistomes of pig farms. Microbiome..

[95] Looft T, Allen HK, Cantarel BL, Levine UY, Bayles DO, Alt DP (2014). Bacteria, phages and pigs: the effects of in-feed antibiotics on the microbiome at different gut locations. ISME J..

[96] Allen HK, Looft T, Bayles DO, Humphrey S, Levine UY, Alt D, Stanton TB. Antibiotics in feed induce prophages in swine fecal microbiomes. mBio. 2011;2(6):e00260–11. 10.1128/mBio.00260-11. 10.1128/mBio.00260-11 PMC3225969 22128350

[97] Kim HB, Borewicz K, White BA, Singer RS, Sreevatsan S, Tu ZJ (2012). Microbial shifts in the swine distal gut in response to the treatment with antimicrobial growth promoter, tylosin. Proc Natl Acad Sci U S A..

[98] Dohoo I, Martin W, Stryhn H (2009). Veterinary epidemiologic research. 2nd edition.

[99] St-Pierre NR (2007). Design and analysis of pen studies in the animal sciences1, 2. J Dairy Sci..

[100] Poulin-Laprade D, Brouard J-S, Gagnon N, Turcotte A, Langlois A, Matte JJ (2021). Resistance determinants and their genetic context in Enterobacteria from a longitudinal study of pigs reared under various husbandry conditions. Appl Environ Microbiol..

