# Supplementary material for: The impacts of viral infection and subsequent antimicrobials on the microbiome-resistome of growing pigs
Source: Microbiome. 2022 Aug 4;10:118. doi: 10.1186/s40168-022-01312-0 (PMC9351240; doi:10.1186/s40168-022-01312-0)
Supplement: Supplementary file 2 — Additional file 1: Figure S1. Boxplots of the raw sequence read counts by time point and treatment group (Minimal—dark grey, Moderate—blue and Intensive—red color). Horizontal lines forming each box represent the first quartile, median and third quartile, while whiskers denote 1.5x the interquartile range. Figure S2. Proportion of host (Sus scrofa) to non-host reads per sample (x-axis) by treatment groups for each time point. Figure S3. Relative abundance of resistance by type (drugs, biocides, multi-compounds and metals) in composite fecal samples, separated by treatment group (panels) and sampling time point (weaning/transport to market). Figure S4. Microbiome composition (NMDS—non metric multidimensional scaling, Bray-Curtis dissimilarity) at phylum level (stress=0.09) by A) sampling time point (ANOSIM—Analysis of similarities P=0.001, PERMANOVA—Permutational multivariate analysis of variance R2=49%, P=0.001). B) by treatment (ANOSIM P=0.238, PERMANOVA R2 <1%, P=0.113). C) microbiome composition at class level (stress=0.10) by sampling time point (ANOSIM P=0.001, PERMANOVA R2=47%, P=0.001). D) by treatment (ANOSIM P=0.388, PERMANOVA R2 <1%, P=0.167). Ellipse indicates 95% confidence interval for distance around centroids of the group. Figure S5. Log-fold change (logFC, x-axes) of microbial phyla for A) each treatment group (Minimal, Moderate and Intensive) comparing sequential sampling time points (i.e., positive logFC values indicate higher abundance in the later time point compared to the earlier time point) and B) between treatment groups (Minimal, Moderate and Intensive) for each sampling time point. Each dot represents a microbial phylum in the group and red dots represent genera that are significantly different between comparison groups (i.e., logFC≥±1, mean abundance ≥ 3, BH adjusted P< 0.05). Figure S6. Log-fold change (logFC, x-axes) of microbial genera for A) each treatment group (Minimal, Moderate and Intensive) comparing sequential time points (i.e., [file 40168_2022_1312_MOESM1_ESM.docx]

**Additional file 1**

**Supplementary Materials**

**(Figure S1-S12 and Table S1-S2)**

**Supplementary Figures**

**
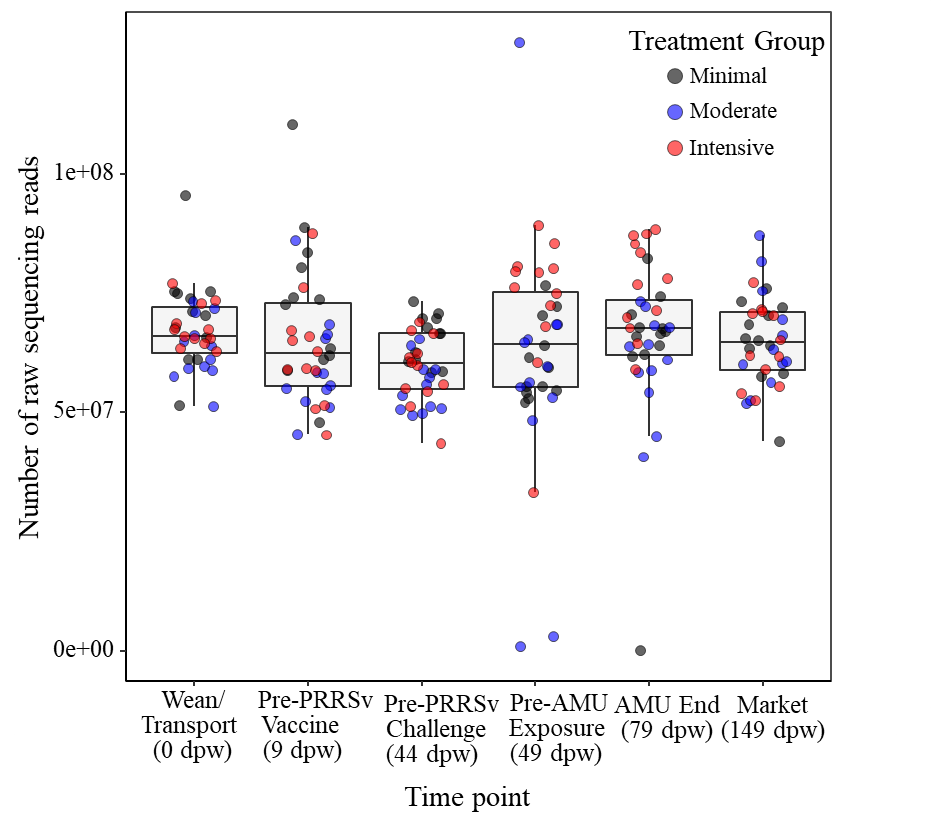
**

**Figure S1.** Boxplots of the raw sequence read counts by time point and treatment group (Minimal—dark grey, Moderate—blue and Intensive—red color). Horizontal lines forming each box represent the first quartile, median and third quartile, while whiskers denote 1.5x the interquartile range.

**
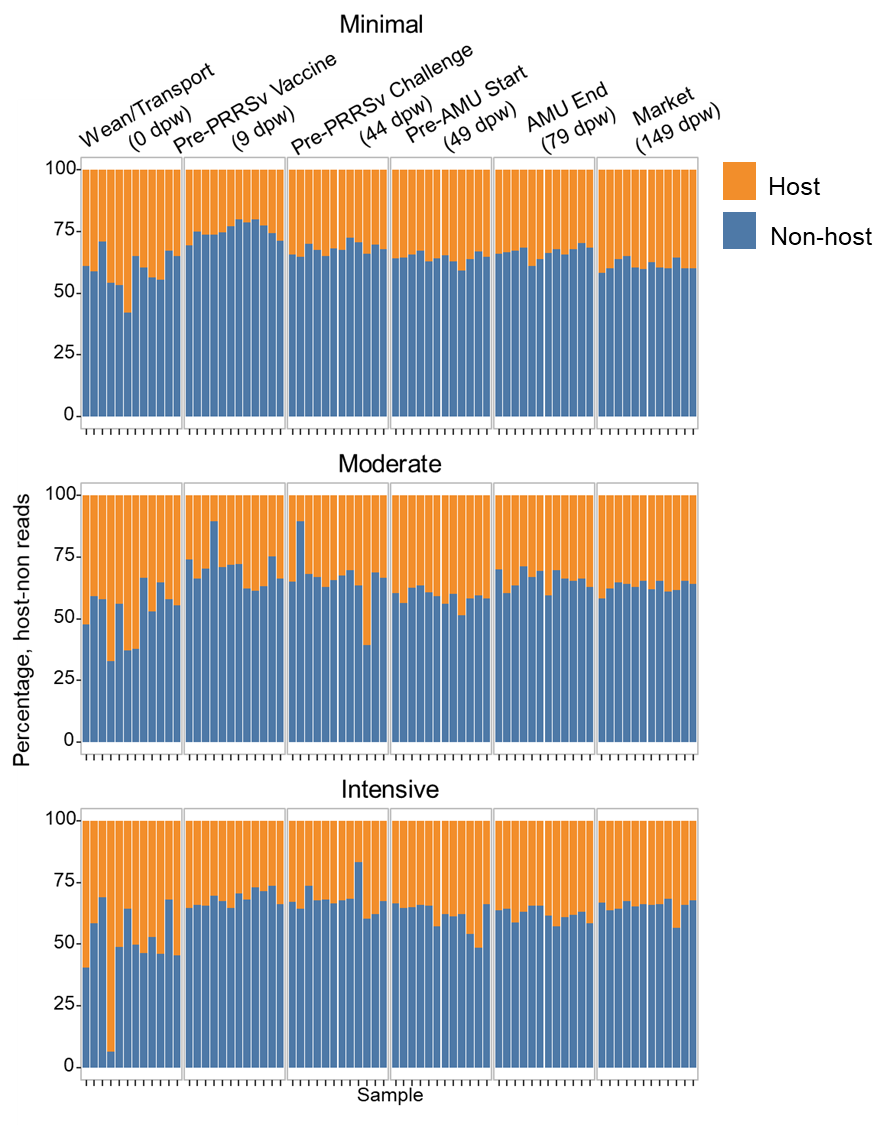
**

**Figure S2.** Proportion of host (*Sus scrofa*) to non-host reads per sample (x-axis) by treatment groups for each time point.

**
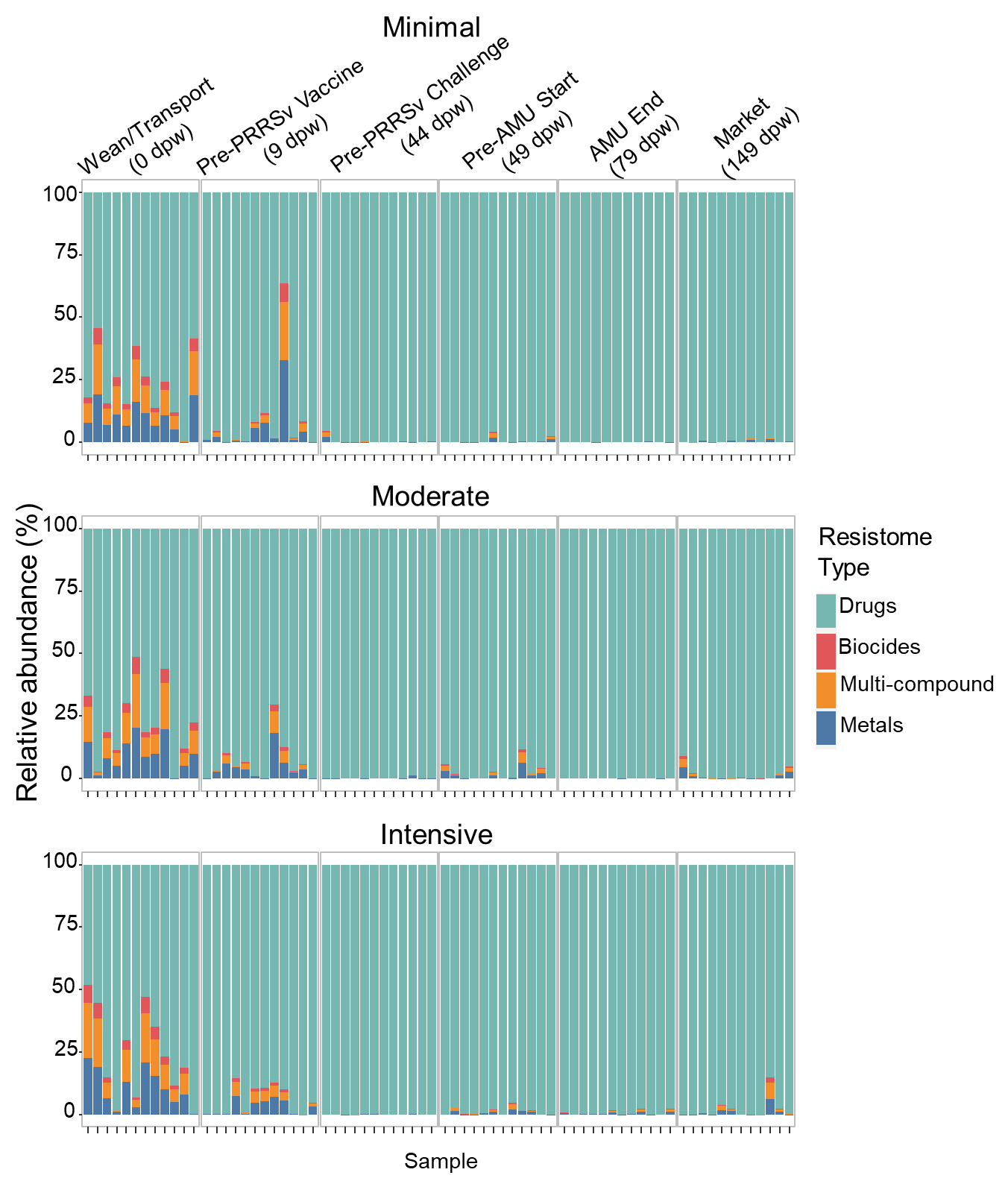
**

**Figure S3.** Relative abundance of resistance by type (drugs, biocides, multi-compounds and metals) in composite fecal samples, separated by treatment group (panels) and sampling time point (weaning/transport to market).

**
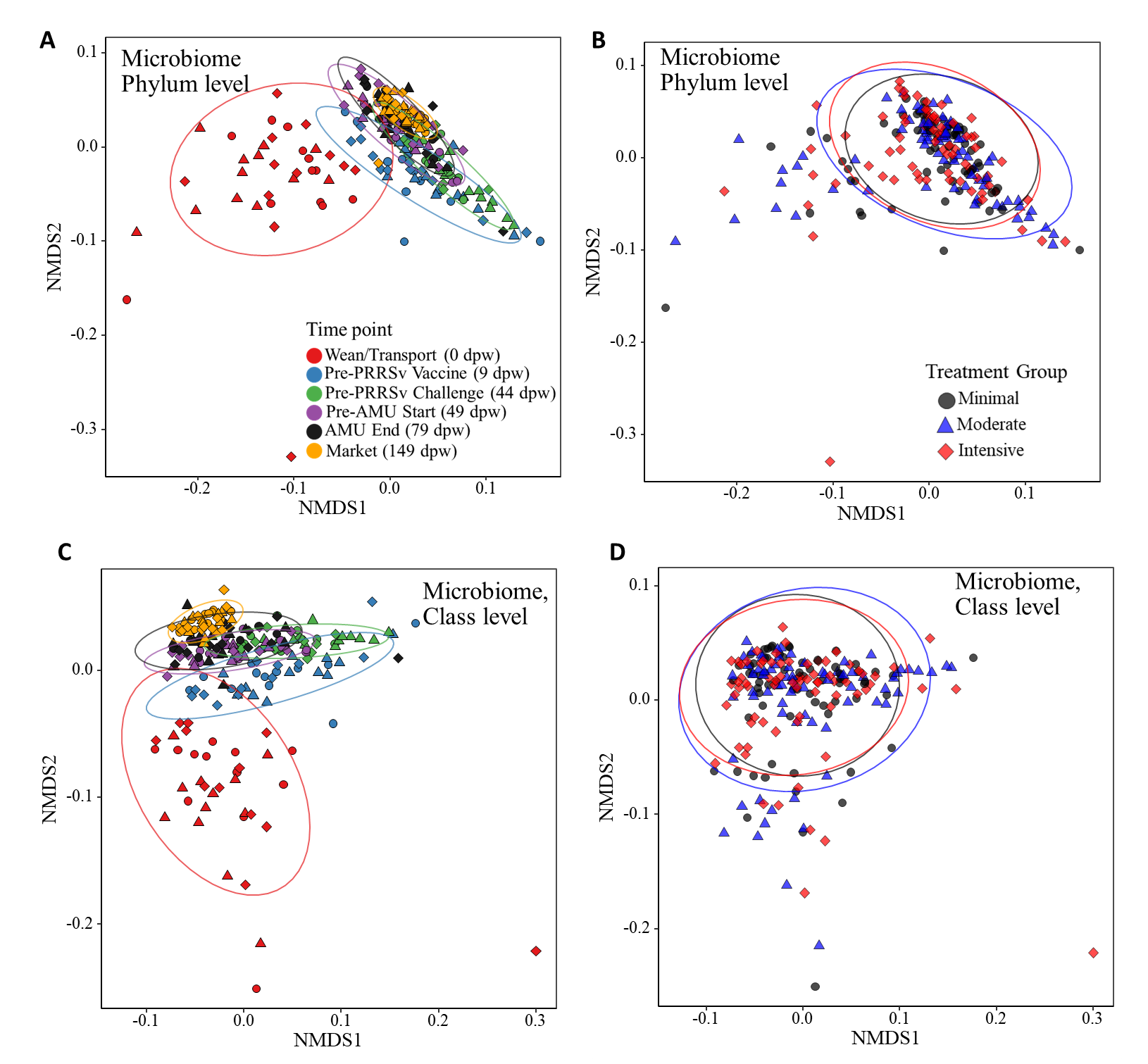
**

**Figure S4.** Microbiome composition (NMDS—non metric multidimensional scaling, Bray-Curtis dissimilarity) at phylum level (stress=0.09) by **A**) sampling time point (ANOSIM*—*Analysis of similarities P=0.001, PERMANOVA—Permutational multivariate analysis of variance R^2^=49%, P=0.001). **B**) by treatment (ANOSIM P=0.238, PERMANOVA R^2^ <1%, P=0.113). **C**) microbiome composition at class level (stress=0.10) by sampling time point (ANOSIM P=0.001, PERMANOVA R^2^=47%, P=0.001). **D**) by treatment (ANOSIM P=0.388, PERMANOVA R^2^ <1%, P=0.167). Ellipse indicates 95% confidence interval for distance around centroids of the group.

**
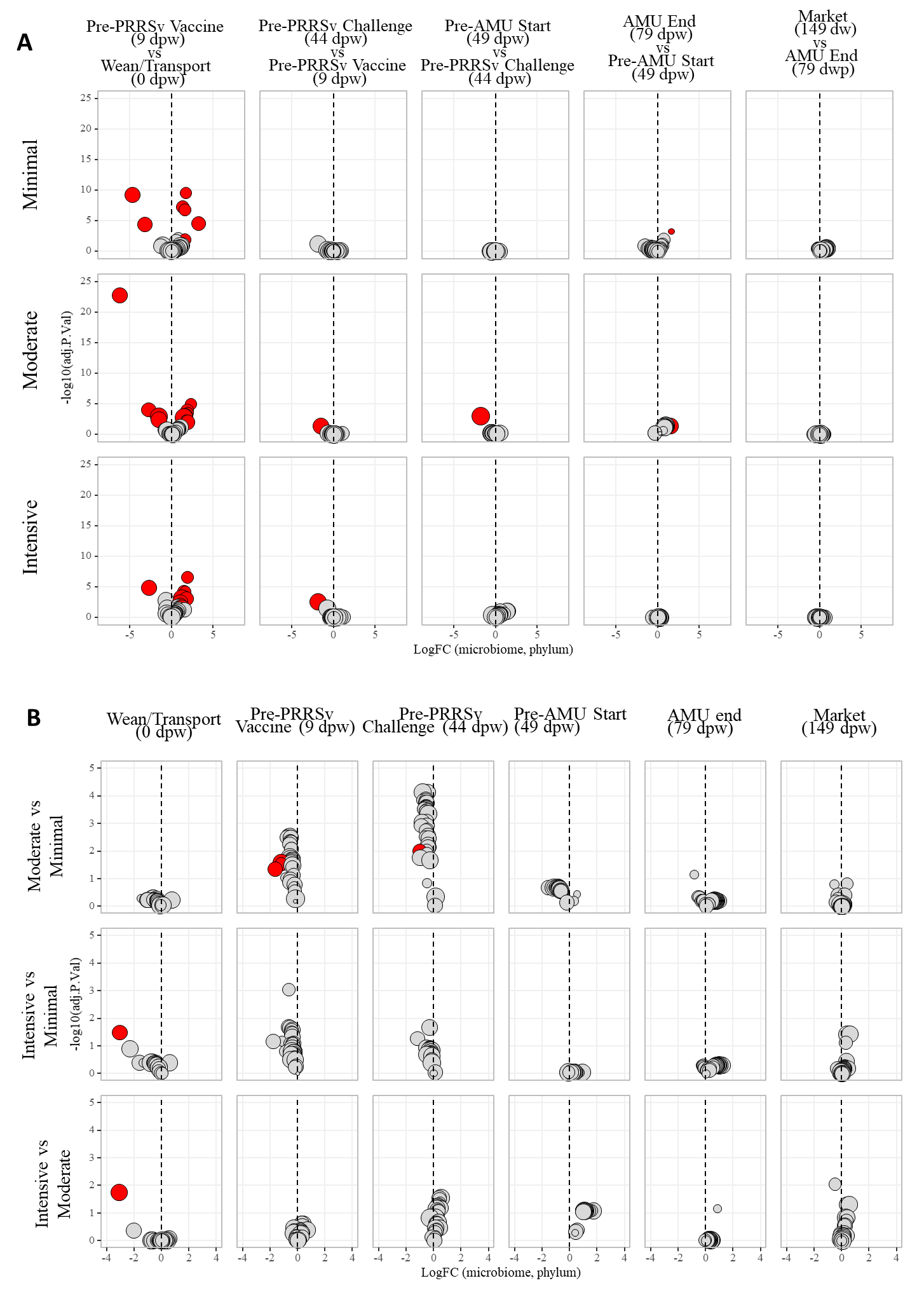
**

**Figure S5.**  Log-fold change (logFC, x-axes) of microbial phyla for **A**) each treatment group (Minimal, Moderate and Intensive) comparing sequential sampling time points (i.e., positive logFC values indicate higher abundance in the later time point compared to the earlier time point) and **B**) between treatment groups (Minimal, Moderate and Intensive) for each sampling time point. Each dot represents a microbial phylum in the group and red dots represent genera that are significantly different between comparison groups (i.e., logFC≥±1, mean abundance ≥ 3, BH adjusted *P*< 0.05).

**
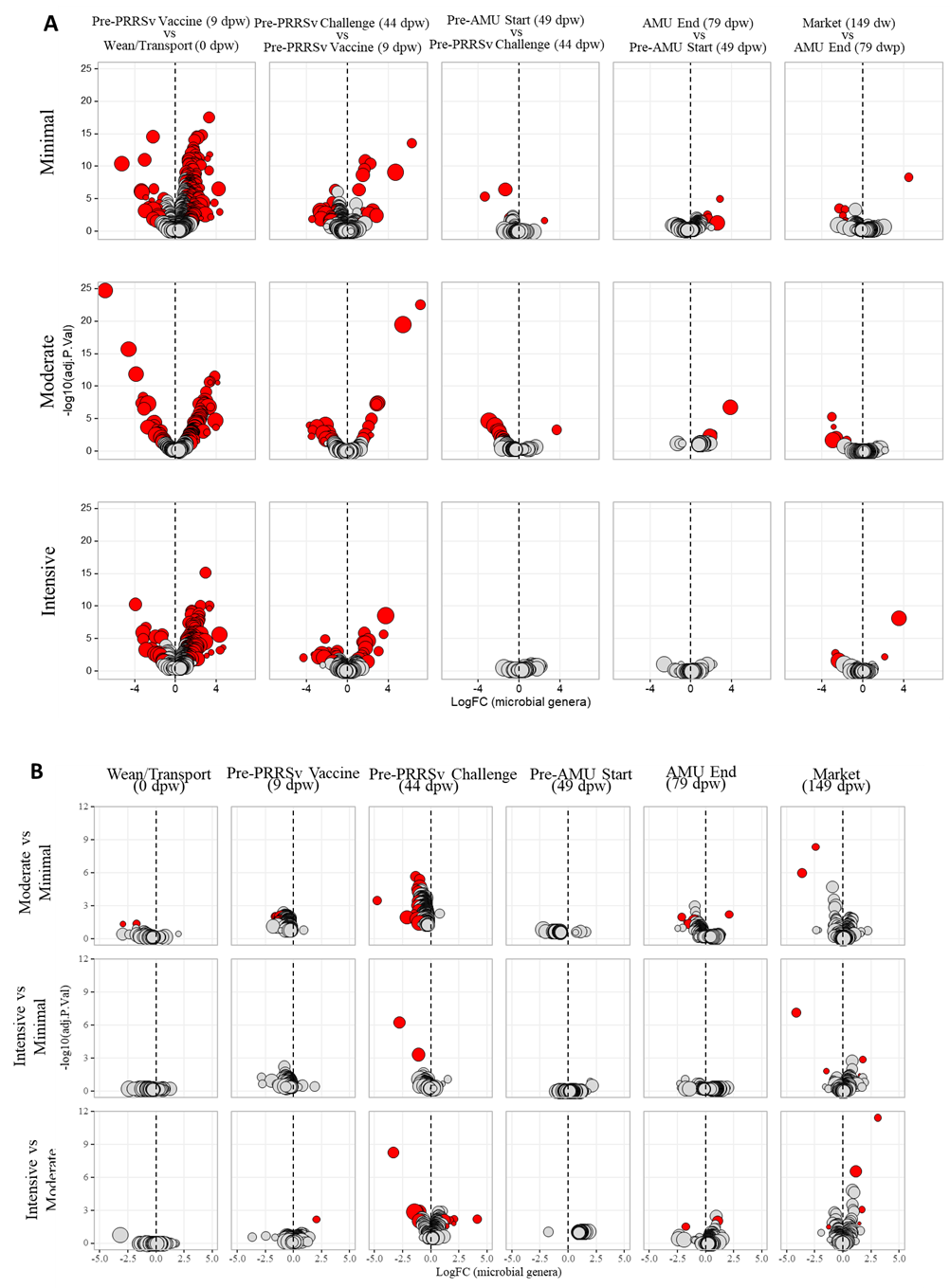
**

**Figure S6.** Log-fold change (logFC, x-axes) of microbial genera for **A**) each treatment group (Minimal, Moderate and Intensive) comparing sequential time points (i.e., positive logFC values indicate higher abundance in the later time point compared to the earlier sampling time point) and **B**) between treatment groups (Minimal, Moderate and Intensive) for each time point. Each dot represents a microbial genera in the group and red dots represent genera that are significantly different between comparison groups (i.e., logFC≥±1, mean abundance ≥ 3, BH adjusted *P*< 0.05).


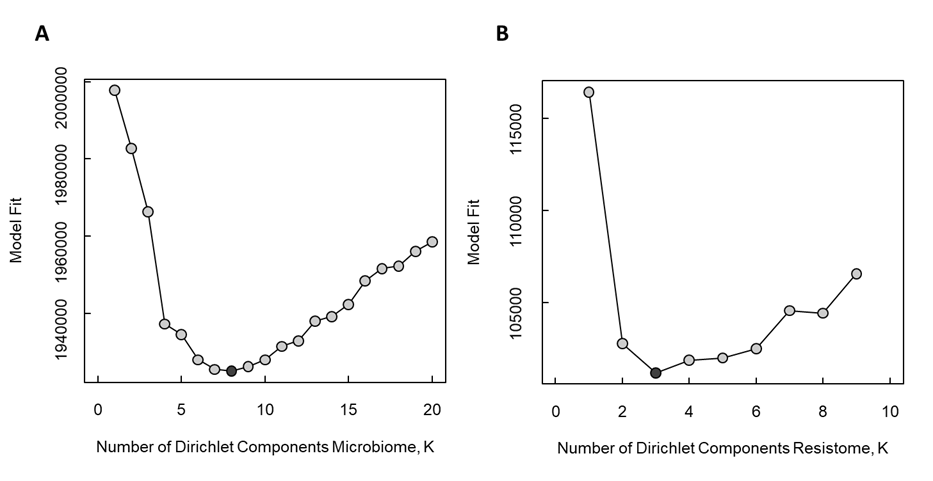


**Figure S7**. Model fit for number of Dirichlet mixture components (DMM), K using the Laplace approximation, **A**) microbiome at genus level and **B**) resistome at ARG level

**
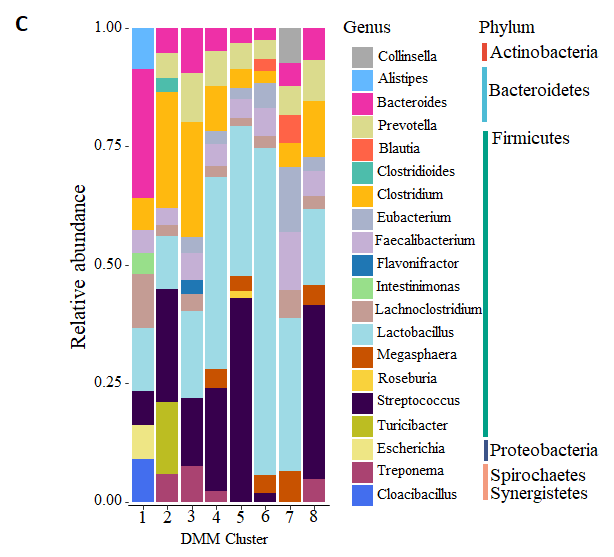
**

**FigureS8.** 100% stacked bar of top 10 taxa (microbial genera) within each DMM cluster.

**
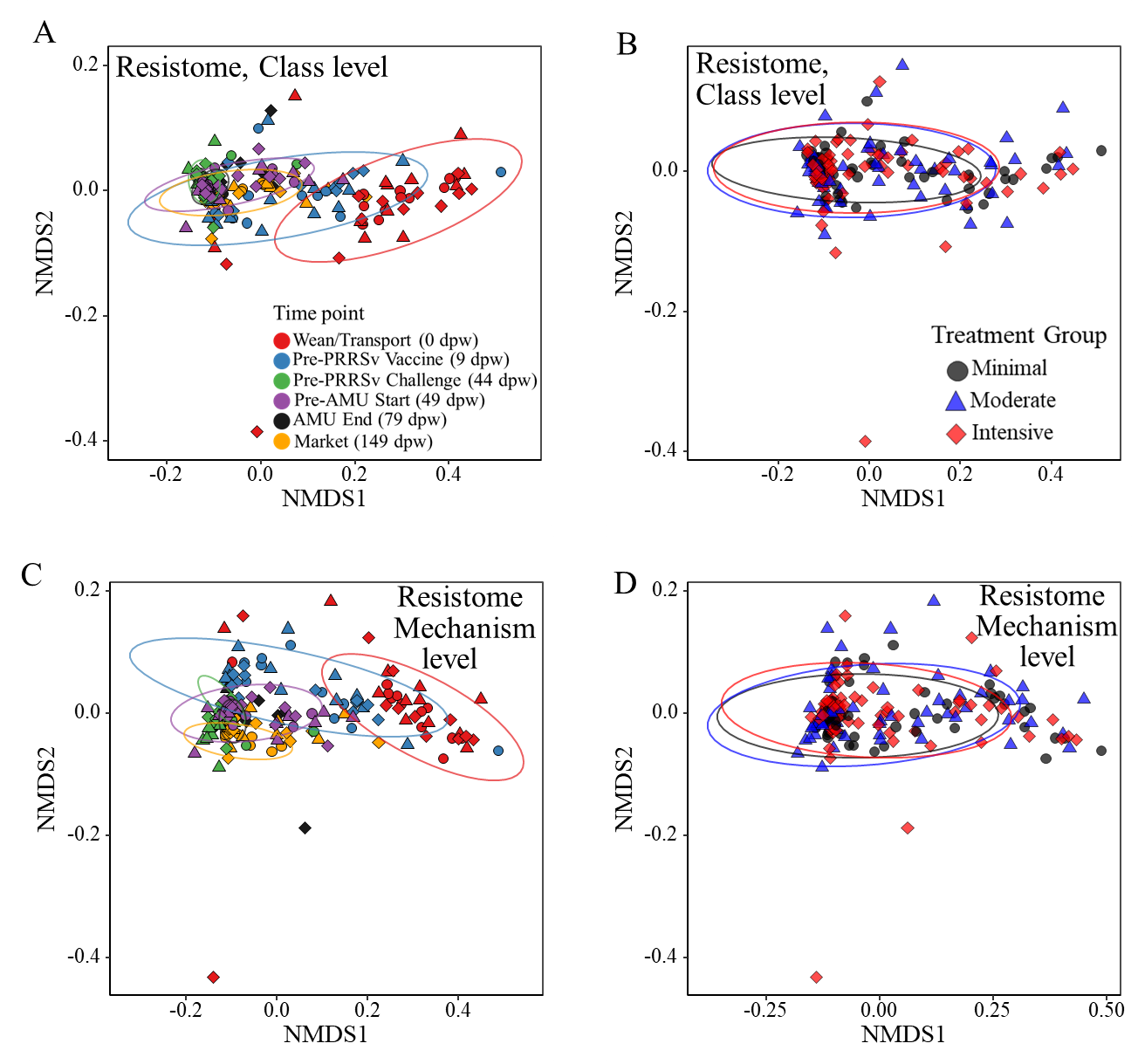
**

**Figure S9.** Resistome composition (NMDS, Bray-Curtis dissimilarity) at class level (stress=0.048) by **A**) sampling time point (ANOSIM *P*=0.001, PERMANOVA R^2^=56%, *P*=0.001). **B**) by treatment (ANOSIM P=0.487, PERMANOVA R^2^<1%, *P*=0.502). **C**) Resistome composition at mechanism level (stress=0.066) by sampling time point (ANOSIM P=0.001, PERMANOVA R^2^=54.8%, *P*=0.001).  **D**) by treatment (ANOSIM *P*=0.08, PERMANOVA R^2^ <1%, *P*=0.344). Ellipse indicates 95% confidence interval for distance around centroids of the group.

**
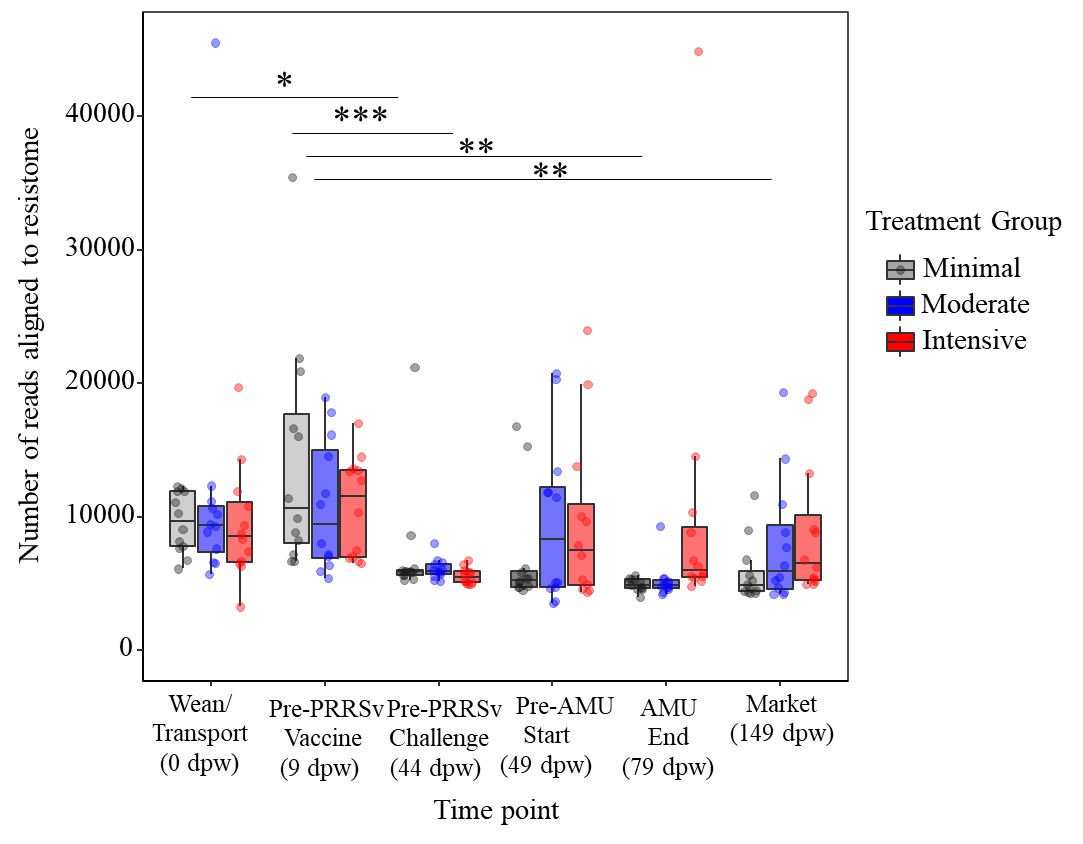
**

**Figure S10.** Number of reads aligned to resistome (i.e ARGs) (cumulative sum scaling normalized) in the MEGARes database by time point and treatment group (represented by colored dots). Horizontal lines forming each box represent the first quartile, median and third quartile, while whiskers denote 1.5x the interquartile range. Each sample is represented by a dot with horizontal jitter.

**
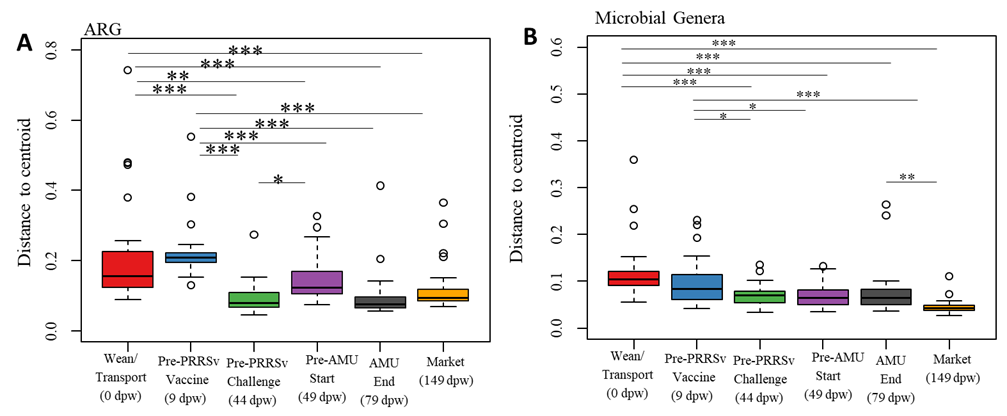
**

**Figure S11.** Effect of sampling time point on the multivariate dispersion of **A**) resistome at ARG level and **B**) microbiome at genus level. Both resistome and microbiome beta-diversity are measured as the distance to their group centroid (using Bray-Curtis distance).


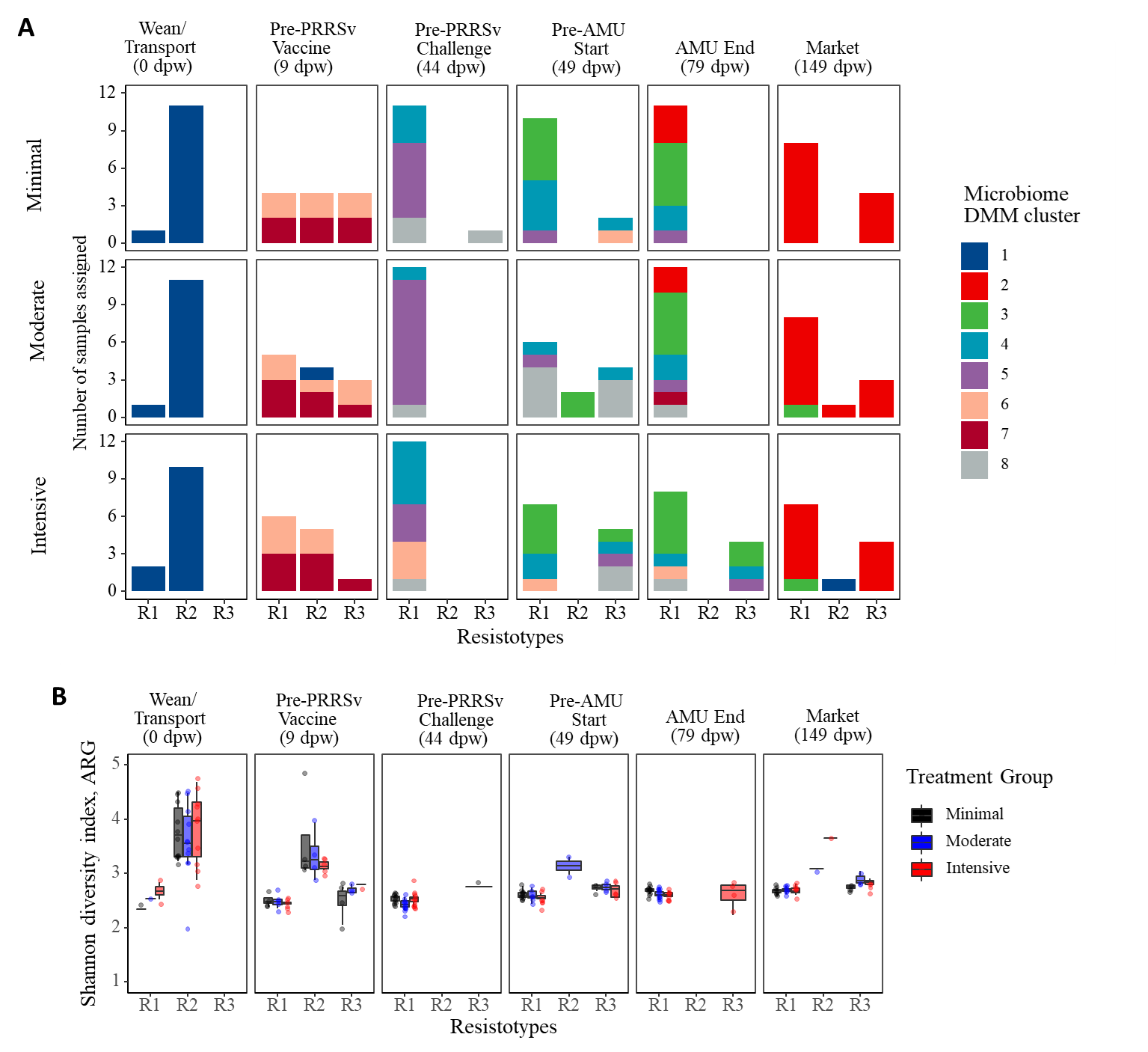


**Figure S12.** Boxplot of **A**) Number of samples for each resistotype depicted as a function of enterotypes (microbiome DMM cluster), **B**) ARG diversity of treatment group for each time point according to the resistotypes.

**Supplementary Tables**

**Table S1**. List of AMR mechanisms for each AMR class presented in heatmap Fig. 7

| **AMR Class** | **AMR Mechanisms** |
| --- | --- |
| Acetate resistance | Acetate resistance protein |
| Acid resistance | Acid resistance protein, Acid resistance regulator |
| Aminoglycosides | 16S rRNA methyltransferases, Aminoglycoside efflux pumps, Aminoglycoside N-acetyltransferases, Aminoglycoside O-nucleotidyltransferases, Aminoglycoside O-phosphotransferases |
| Arsenic resistance | Arsenic resistance membrane transporter, Arsenic resistance protein, Arsenic resistance regulator |
| Bacitracin | Undecaprenyl pyrophosphate phosphatase |
| *β*-lactamas | Class A betalactamases, Class B betalactamases, Class C betalactamases, Class D betalactamases, Mutant porin proteins, Penicillin binding protein |
| Biguanide resistance | Biguanide cation efflux |
| Biocide and metal resistance | Biocide and metal ABC efflux pumps, Biocide and metal resistance protein |
| Cationic antimicrobial peptides | Defensin-resistant mprF, Lipid A modification |
| Chromium resistance | Chromium resistance protein |
| Copper resistance | Copper resistance protein, Copper resistance regulator |
| Drug and biocide and metal resistance | Drug and biocide and metal RND efflux pumps, Drug and biocide and metal RND efflux regulator |
| Drug and biocide resistance | Drug and biocide ABC efflux pumps, Drug and biocide MATE efflux pumps, Drug and biocide MFS efflux pumps, Drug and biocide MFS efflux regulator, Drug and biocide RND efflux pumps, Drug and biocide RND efflux regulator,Drug and biocide SMR efflux pumps |
| Fluoroquinolones | Quinolone resistance protein Qnr |
| Fosfomycin | Fosfomycin thiol transferases |
| Glycopeptides | Bleomycin resistance protein, VanB-type accessory protein, VanB-type regulator, VanB-type resistance protein, VanD-type accessory protein, VanD-type regulator, VanD-type resistance protein, VanG-type accessory protein, VanG-type resistance protein |
| Iron resistance | Iron resistance protein |
| Mercury resistance | Mercury resistance protein, Mercury resistance regulator |
| Metronidazole | nim nitroimidazole reductase |
| MLS | 23S rRNA methyltransferases, Lincosamide nucleotidyltransferases, Macrolide phosphotransferases, MLS resistance ABC efflux pumps, MLS resistance MFS efflux pumps, Streptogramin A O-acetyltransferase |
| Multi-biocide resistance | Multi-biocide resistance protein, Multi-biocide resistance regulator, Multi-biocide RND efflux pump, Multi-biocide SMR efflux pump |
| Multi-drug resistance | Multi-drug ABC efflux pumps, Multi-drug MFS efflux pumps, Multi-drug RND efflux pumps,Multi-drug RND efflux regulator |
| Multi-metal resistance | Multi-metal ABC efflux pumps, Multi-metal resistance protein, Multi-metal resistance regulator, Multi-metal RND efflux pumps, Multi-metal RND efflux regulator |
| Nickel resistance | Nickel ABC efflux pumps, Nickel ABC efflux regulator |
| Nucleosides | Streptothricin acetyltransferase |
| Peroxide resistance | Peroxide resistance protein, Peroxide resistance stress protein |
| Phenicol | Chloramphenicol acetyltransferases, Phenicol resistance MFS efflux pumps |
| Phenolic compound resistance | Phenolic resistance protein |
| Quaternary Ammonium Compounds (QACs) resistance | QAC efflux pump |
| Sodium resistance | Sodium resistance protein |
| Sulfonamides | Sulfonamide-resistant dihydropteroate synthases |
| Tellurium resistance | Tellurium resistance protein |
| Tetracyclines | Tetracycline inactivation enzymes, Tetracycline resistance MFS efflux pumps, Tetracycline resistance ribosomal protection proteins |
| Trimethoprim | Dihydrofolate reductase |
| Zinc resistance | Zinc resistance protein, Zinc resistance regulator |

**Table S2**. List of low abundant ARGs that were detected post-PRRSv challenge in moderate and intensive treatment groups.

| **AMR class** | **Low abundance ARGs present in moderate and intensive treatment groups post-PRRSv challenge** |
| --- | --- |
| Zinc resistance | *zitB, znuA, znuC, zraR, zuR* |
| Trimethoprim | *dfra* |
| Tellurium resistance | *terD, terW, terZ* |
| Sodium resistance | *chaA, nhaA, nhaB* |
| Phenolic compound resistance | *fabI* |
| Peroxide resistance | *ibpA, ibpB, sodA, sodB, ydeI* |
| Nickel resistance | *nikE, nikR* |
| Multi-metal resistance | *arscM, cadX, corA, corB, corC, corD, cusA, cusB, cusC, cusR, cusS, dsbA, dsbB, dsbC, fecD, fecE, fieF, mgtA, mntH, mntP, mntR, modA, modB, modC, pcoE, pitA, rcnA, rcnB, rcnR, silA, silB, silC, silE, silP, silS, ygjH, zinT, zntazraS* |
| Multi-drug resistance | *asmA* |
| Multi-biocide resistance | *ariR, oxyrkP,rpoS, soxrB, sugE,tolC, ydeO* |
| Mercury resistance | *merC, merr1, merT* |
| Iron-resistance | *ybtP, ybtQ* |
| Glycopeptides | *vanxyG* |
| Drug and biocide resistance | *acrA, acrB,acrE,acrF, acrS, bcR,cpxaR,emrA, emrB, emrD, emrK, emrY, evgS, gadW, gadX, kpN, marA, marR, mdfA, mdtE, mdtF, mdtG, mdtH, mdtI, mdtJ, mdtK, mdtM, mdtN, mdtO, mdtP, mvrC, qacedelta1, yogI* |
| Drug and biocide and metal resistance | *acrD, baeR, baeS, cmeD, cmeE, mdtA, mdtB, mdtC, robA* |
| Copper resistance | *bhsA, comR, copA, cueO, cueR, cutA, cutC, cutE, cutF, pcoA, pcoB, pcoC, pcoD, pcoR, pcoS* |
| Cationic antimicrobial peptides | *eptA, pmrF, ugD* |
| Biocide and metal resistance | *fetA, fetB, glpF, tehB, ychH, ygiW, yhcN, yieF* |
| β-lactamas | *ampC, ampH, blaeC, pbp2, pbp4b* |
| Bacitracin | *bacA* |
| Arsenic resistance | *acr3, pstB, pstC, pstS* |
| Acid resistance | *asR,evgA, gadA, gadB, gadC, gadE, ydeP* |
| Acetate resistance | *iclR, lpdT* |
